# Toxicity Assessment of Long-Term Exposure to Non-Thermal Plasma Activated Water in Mice

**DOI:** 10.3390/ijms222111534

**Published:** 2021-10-26

**Authors:** Valentin Nastasa, Aurelian-Sorin Pasca, Razvan-Nicolae Malancus, Andra-Cristina Bostanaru, Luminita-Iuliana Ailincai, Elena-Laura Ursu, Ana-Lavinia Vasiliu, Bogdan Minea, Eugen Hnatiuc, Mihai Mares

**Affiliations:** 1 Laboratory of Antimicrobial Chemotherapy, Faculty of Veterinary Medicine, “Ion Ionescu de la Brad” University of Life Sciences (IULS), 8 Mihail Sadoveanu Alley, 700489 Iasi, Romania; vnastasa@uaiasi.ro (V.N.); spasca@uaiasi.ro (A.-S.P.); razvanmalancus@uaiasi.ro (R.-N.M.); acbostanaru@uaiasi.ro (A.-C.B.); lailincai@uaiasi.ro (L.-I.A.); ehnatiuc@yahoo.fr (E.H.); mmares@uaiasi.ro (M.M.); 2 “Petru Poni” Institute of Macromolecular Chemistry, 41A Aleea Grigore Ghica-Voda, 700487 Iasi, Romania; ursu.laura@icmpp.ro (E.-L.U.); vasiliu.lavinia@icmpp.ro (A.-L.V.); 3 Department of Surgery, Faculty of Dental Medicine, “Grigore T. Popa” University of Medicine and Pharmacy of Iasi, 16 Universitatii Street, 700115 Iași, Romania

**Keywords:** non-thermal plasma activated water (PAW), toxicity in mice, GlidArc reactor, immunohistochemistry, cytokine profile

## Abstract

Non-thermal plasma activated water (PAW) has recently emerged as a powerful antimicrobial agent. Despite numerous potential bio-medical applications, studies concerning toxicity in live animals, especially after long-term exposure, are scarce. Our study aimed to assess the effects of long-term watering with PAW on the health of CD1 mice. PAW was prepared from distilled water with a GlidArc reactor according to a previously published protocol. The pH was 2.78. The mice received PAW (experimental group) or tap water (control group) daily for 90 days as the sole water source. After 90 days, the following investigations were performed on the euthanatized animals: gross necropsy, teeth mineral composition, histopathology, immunohistochemistry, hematology, blood biochemistry, methemoglobin level and cytokine profile. Mice tolerated PAW very well and no adverse effects were observed during the entire period of the experiment. Histopathological examination of the organs and tissues did not reveal any structural changes. Moreover, the expression of proliferation markers PCNA and Ki67 has not been identified in the epithelium of the upper digestive tract, indicating the absence of any pre- or neoplastic transformations. The results of our study demonstrated that long-term exposure to PAW caused no toxic effects and could be used as oral antiseptic solution in dental medicine.

## 1. Introduction

In physics, the term “plasma” refers to the fourth state of aggregation of matter that consists of charged species (ions and free electrons) that reach temperatures as high as 10,000 K; it exists in nature in a wide variety of forms and it can be artificially created in different ways, grouped in technology as thermal-type and non-thermal type plasma. In non-thermal plasma, only the electrons reach high temperatures, while the ions (which make up the majority of the mass) remain close to room temperature, which renders the plasma itself much colder, often close to room temperature (cold plasma). Due to the diversity of fields of application (industrial, bio-medical) the generation of a certain type of plasma and the specific requirements of each application (temperature, potential, chemical composition, flow, etc.) depend on the selection of the plasma source. Technologically, the most commonly used method of generating plasma at low temperatures is to apply an electric field in a neutral gas [1].

Non-thermal plasma generated in atmospheric conditions (CAP) is of particular interest because it can be produced with simple plasma sources, including corona discharges, glow discharges, dielectric barrier discharges, GlidArc and plasma jets [2]. Due to its chemical composition, rich in reactive oxygen and nitrogen species (RONS) [3,4,5,6], non-thermal plasma has many biomedical applications in dermatology [7,8,9], microbiology and many other fields [10,11,12].

Research on non-thermal plasma (cold plasma) in biology and medicine is focused on its use in both direct and indirect treatments. Direct treatments imply a direct contact with charged plasma particles, while indirect treatments use plasma “activated” gases or liquids [13]. In recent years, special emphasis was placed on the interaction of plasma with various media, particularly liquids (water, saline solution, Ringer-lactate solution, culture media) addressing various fields of application in agriculture and bio-medicine [14,15,16,17,18,19,20,21,22,23,24,25,26]. Compared to applying plasma directly on living tissues, plasma activated media (PAM) have the advantage of longer term storage (up to 30 days at −80 °C), depending on the medium and storage conditions [27], although over time all plasma-treated solutions lose their reactivity (PAW aging) [5].

Treating water with non-thermal plasma induces structural changes (e.g., breaking the hydrogen bonds generates mono-molecular water) and produces numerous RONS. The primary reactive species generated during the process (atomic oxygen, singlet oxygen, superoxide, ozone, hydroxyl radicals and atomic nitrogen) continue to interact with the surrounding environment, forming secondary reactive species (hydrogen peroxide, peroxynitrite, nitric oxide, nitrates and nitrite ions) which subsequently dissolve in water [5,6,14,15,25,28,29].

Tarabová et al. demonstrated that the presence of nitrites (NO_2_^−^) and hydrogen peroxide (H_2_O_2_) in PAW led predominantly to the production of peroxynitrous acid [6]. The most important active species are hydrogen peroxide and peroxynitrite, both with strong antimicrobial activity. This has implications for the medical field, especially for decontamination and sanitation [14,15,25,30,31,32,33,34,35,36,37]. Many studies report antimicrobial effects of PAW on bacteria [38,39,40,41,42], and their resistant structures (spores) [43]. PAW was also used with very good results in agriculture to stimulate seed germination [44,45] and plant growth [46], as well as in the food industry for the decontamination of fruits, vegetables and other items [47].

Although there are many studies describing the interaction of plasma and biological organisms, there is little research that assesses the in vivo toxicity of CAP or PAM, particularly over the long-term. Various articles report levels and mechanisms of wanted direct or indirect toxicity against microbial cells and viruses [48,49,50,51,52], algal cells [53], and various tumor cell lines [54,55,56,57,58,59,60,61,62]. Some studies report a lack of cytotoxicity on normal cells [63] others, on the contrary, report the presence of cytotoxicity (that can be dose dependent) [60,61,62,64,65,66]. Much of this research consists of in vitro studies. In vivo toxicity studies on mammals are scarce and most involve short-term contacts (less than 24 h) with CAP or PAM [67,68,69,70]. A lack of toxicity is reported.

Cytotoxicity mechanisms include damage to cell walls, cell membranes, DNA, enzymes and other proteins [50,51,54]. The mechanisms are diverse but they all seem to be caused by the generated RONS. Furthermore, at the cell level, endogenous RONS are important components of intracellular signaling cascades. Therefore, a surplus of exogenous RONS can have a detrimental effect on the health of various biological organisms, not only by inducing oxidative stress, but also by disturbing the delicate balance of the complex network of intracellular biochemical pathways [61,71,72,73]. Some authors even report a certain level of selective toxicity against procaryotic cells (bacteria), compared to eucaryotic cells, because the later have protective mechanisms against RONS [54,74].

Because of the limited toxicological data offered by short-term exposure studies, the safety of PAW interaction with living mammals cannot be considered as thoroughly assessed. Because of its potential to be used as an oral antiseptic solution or for other dental applications [75,76], which can lead to accidental ingestion that may have consequences for human health, our study aimed to evaluate the in vivo long-term toxicity of PAW, in CD1 mice, by administering it daily for 90 days.

## 2. Results

### 2.1. Effect of Long-Term PAW Consumption on Vital Teeth: EDX, SEM and AFM Data

We investigated to what extent PAW could affect the mineral composition and surface micro-morphology of vital mouse teeth by long-term exposure. The EDX analysis showed that after 90 days of PAW consumption there were no significant differences (*p* > 0.05) of mineral composition (% atoms) between the two groups, except for potassium (Table 1). The data recorded in this case showed a significant increase (*p* = 0.0125) of this mineral in the experimental group. There were also minor differences in ion composition, but without clinical significance; diet may have been a factor that influenced these variations. In the experimental group there were increases for the following elements: O (+4.43%), F (+0.06%), Na (+0.07%), Mg (+0.13%), Al (+0.07%), K (+0.04%), Ca (+2.68%), P (+1.87%) and decreases for C (−7.8%) and N (−1.91%).

**Figure 1:**
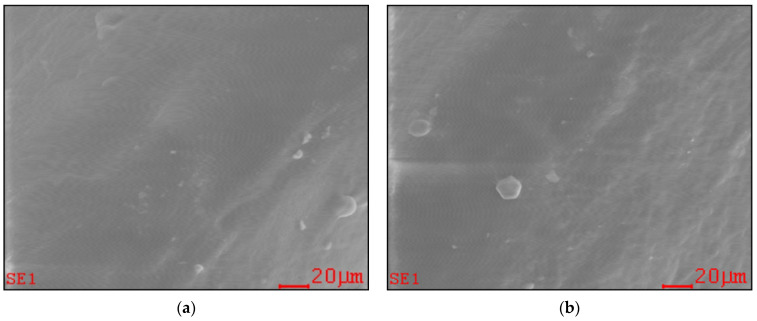
Tooth surface micro-morphology (SEM): (**a**) mouse from the control group; (**b**) mouse from the PAW treated group.

The SEM investigations showed no substantial modifications of teeth surface micro-morphology in the PAW treated group compared to the control group (Figure 1).

**Figure 2:**
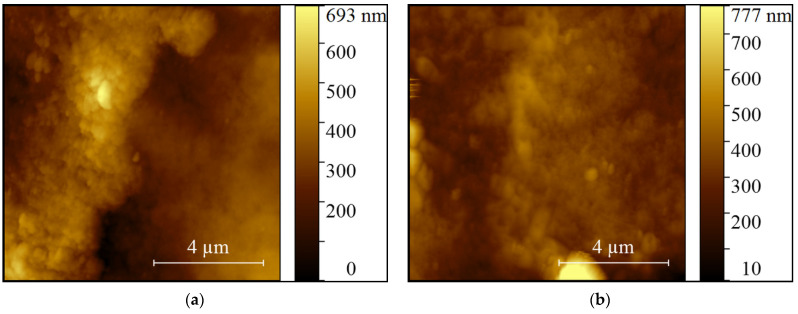
AFM topography of teeth surface: (**a**) mouse from the control group; (**b**) mouse from the PAW treated group.

AFM images shown in Figure 2 reveal the presence of granular structures on the teeth surface for the PAW treated group and the control group. Comparing images of the surfaces of teeth from both groups, no significant surface alterations were observed.

### 2.2. The Effects of Long-Term PAW Consumption on Vital Organs

#### 2.2.1. Weight

The weight of organs that are essential for maintaining body homeostasis (heart, spleen, liver, kidneys, brain) was measured (Table 2). The analyzed organs did not show significant differences in weight between the two groups of mice (*p* > 0.05), with the exception of the spleen that was smaller in the PAW group, both in terms of absolute (*p* = 0.01) and relative weight (*p* = 0.005).

#### 2.2.2. Histopathology and Immunohistochemistry

The microscopic examination of the digestive system—tongue (Figure 3a), oral mucosae (Figure S1), esophagus (Figure 3b), gastric mucosae (Figure 3c, Figures S2 and S3), duodenum, jejunum, colon, liver, pancreas and sublingual glands (Figures S4–S9)—showed no morphological or histological changes.

The histology of the heart (myocardium), large blood vessels (coronary artery), lung, cerebral cortex, cerebellum, kidneys (Figures S10–S16) and of the adrenal glands (Figures S17 and S18) and the lymphatic system—spleen (Figure 3d) and lymph node (Figure S19) was also normal.

Immunohistochemical examination of mice epithelia that came in direct and prolonged contact with PAW (tongue, esophagus and stomach) showed no pre- or neoplastic transformation. The expressions of the Ki-67 and PCNA proliferation markers were absent in the tongue (Figure 4a,b) and at low levels in the esophagus (Figure 4c,d) and the basal layer of the stomach squamous epithelium (Figure 4e,f), consistent with the normal limits of a physiological turn-over.

### 2.3. The Effects of Long-Term PAW Consumption on Blood Parameters

The health status of CD1 mice was further assessed by measuring hematological and biochemical parameters, as well as methemoglobin (MetHb) levels.

#### 2.3.1. Hematological Parameters

Mean values of hematological parameters are presented in Table 3. The hematological parameters in the PAW group did not show any significant differences compared to the control group.

#### 2.3.2. Biochemical Parameters

The values of the biochemical parameters in mice from the two groups are presented in Table 4. There were no significant differences in blood biochemistry between the control group and the PAW group (*p* >0.05).

#### 2.3.3. Methemoglobin (MetHb) Levels

The values recorded in the two groups were 3.63 ± 0.46 in the control group, watered with tap water, and 3.70 ± 0.33 in the experimental group, watered with PAW. MetHb levels recorded in the experimental group did not differ significantly from those recorded in the control group (*p* = 0.81). The averages of these measurements were below the clinically tolerated human minimum of 5%, a level from which adverse health effects can be observed. No adverse effects were observed during the 90 days of the experiment.

### 2.4. Effects of Long-Term PAW Consumption on the Immune Response

Circulation levels of cytokines were determined to verify if long-term PAW consumption caused an inflammatory status in CD1 mice (Table 5). The data obtained showed no significant differences (*p* > 0.05) between the control group and the PAW group for the analyzed cytokines.

## 3. Discussion

The most important factor involved in assessing the toxicity of repeated administration of new molecules with therapeutic potential in vivo is the evaluation of the effects on organs that are essential for maintaining body homeostasis (heart, spleen, liver, kidneys, brain). Necropsy, histopathological examinations, hematology and blood biochemistry analyses are fundamental steps in toxicological research, used to validate new substances with therapeutic potential. Along with the macroscopic and histological examination of the organs, measuring their weight is the most sensitive indicator of the effect of PAW, because significant differences in organ weight can appear in the absence of morphological changes.

**Figure 3:**
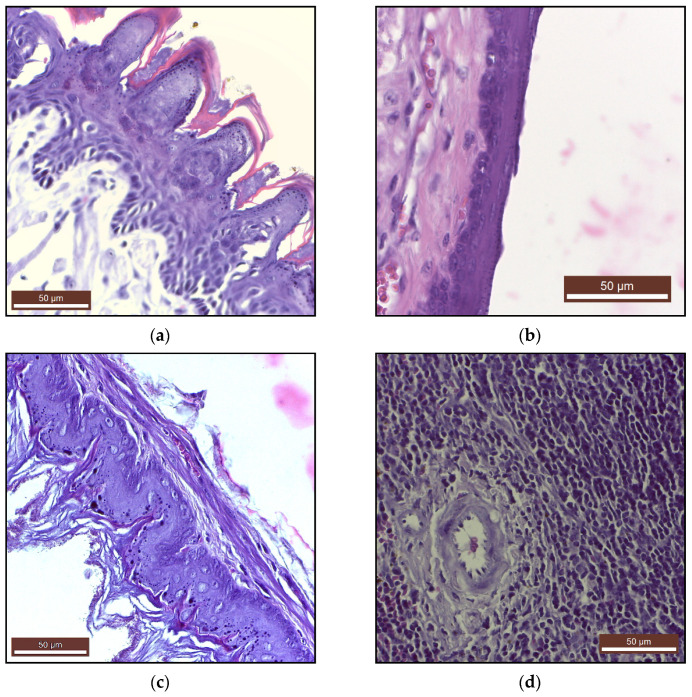
Tissue sections (Masson’s trichrome staining) of: (**a**) tongue, (**b**) esophagus, (**c**) keratinized gastric mucosa, (**d**) spleen.

The long-term health effects of PAW are not described in the scientific literature. There are a few publications that discuss the acute toxicity of PAW [69,70]. A study of nude BALB/c immunodeficient mice by XU et al., where PAW oral lavage was administered to the mice daily for 2 weeks, concluded that PAW did not negatively impact the weight and structure of major organs, hematological parameters or blood biochemistry, and it did not cause any other signs of acute toxicity [70]. Our study reports a similar lack of toxicity (Table 2, Table 3 and Table 4, Figure 3 and Figures S1–S19), but following an actual ingestion of PAW, which had, therefore, a much longer and deeper interaction with the mammalian organism. The administration was also much more extensive (90 days). In general, the PAW treated mice in our study did not show significant differences in organ weight, compared to the control group, with the exception of the spleen. In this latter case, however, necropsy, histology and immunohistochemistry investigations discovered no pathological alterations in the organ, despite these weight differences. Furthermore, hematological, biochemical and immunological assessments did not show modifications that would support the presence of pathological processes. This phenomenon needs further investigation in future studies.

In our study, no changes in shape, consistency and volume were observed on macroscopic examination of vital organs. Histological examination did not reveal the existence of morphological changes that would endanger the health of mice after daily consumption of PAW for 90 days. Histological and immunohistochemical examination of the tongue, oral mucosa, gastric mucosa, duodenum, jejunum, colon, liver, pancreas and sublingual glands in the long-term administration of PAW to mice did not reveal any inflammatory transformation of pre- or neoplastic nature, and no changes in intestinal function were observed clinically.

**Figure 4:**
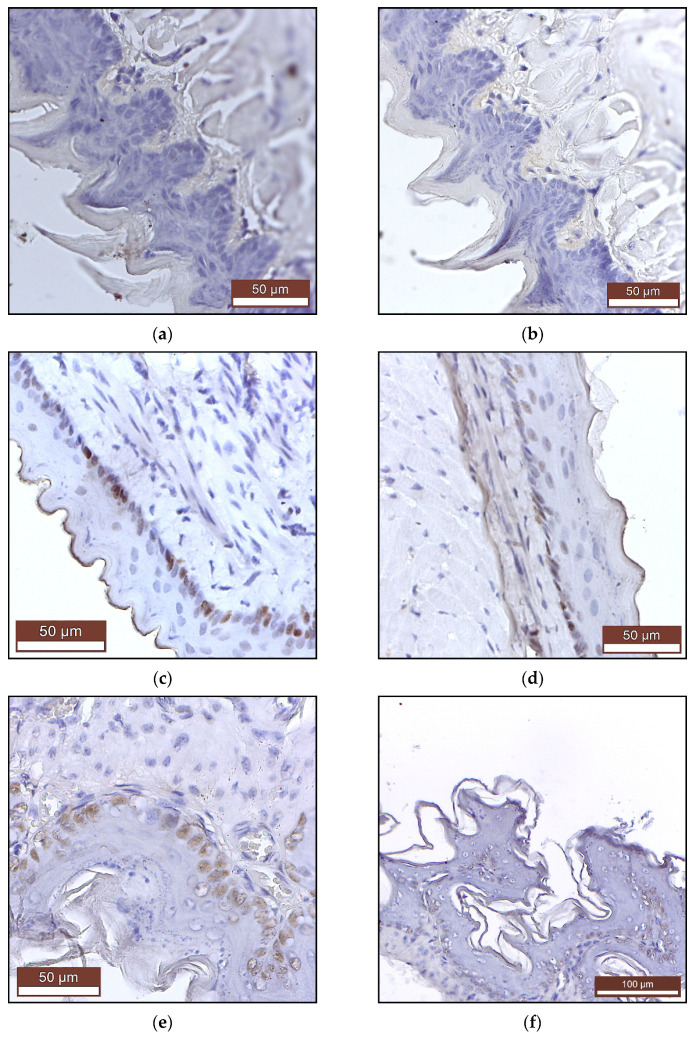
Immunohistochemistry—signals of Ki-67 and PCNA proliferation markers: (**a**) tongue mucosa—Ki-67, (**b**) tongue mucosa—PCNA, (**c**) esophagus mucosa—Ki-67, (**d**) esophagus mucosa—PCNA, (**e**) gastric keratinized mucosa—Ki-67, (**f**) gastric keratinized mucosa—PCNA.

The potential acute and sub-acute toxicity of orally administrated PAM was investigated by Han et al. Experimentally, it was shown that rats given a single dose of 5000 mg/kg of a soy based edible film treated for 15 min with non-thermal plasma showed no signs of acute toxicity or death after 14 days of monitoring. There were also no signs of subacute toxicity or death in rats following repeated administration of 1000 mg/kg/day for 14 days of the same nutritional film [77]. Compared to this study, in our experimental design PAW interacts directly with the digestive mucosa and with the contents of the stomach and the intestine, which would make a potential toxicity much more likely to manifest itself. Nevertheless, our histological (Figure 3a–c) and immunohistochemical (Figure 4) analyses of oral, esophageal and gastric mucosae showed no signs of toxicity.

Clinical signs of toxicity such as color changes of the skin or mucous membranes, piloerection, diarrhea, weight loss, behavioral changes (reduced food intake, reduced locomotor activity, abnormal body posture) were also not present.

XU et al. reported a lack of lethal effects or signs of acute toxicity in rabbits that received one injection of a plasma activated liquid in their bone marrow [69]. Modifications of the percentages of lymphocytes and neutrophiles were however observed. Our data did not reveal adverse effects on major blood indicators to be caused by long-term PAW consumption (Table 3). Additionally, there were no significant changes in the general immunological status of the mice (Table 5). Interleukins, especially those with a proinflammatory role and involved in maintaining homeostasis at the digestive level (IL-1, IL-6, IL- 12p40, IL-12p70, IL-13, IL17a, IFNγ, IL-22, GM-CSF, TNFα), showed no significant differences (*p* > 0.05) compared to the mice in the control group.

The difference was probably due to the different location of PAM contact, the bone marrow, which is the source of both the lymphoid and the myeloid cell lines and, therefore, has a major impact on the levels of lymphocytes and neutrophiles.

We also report a lack of methemoglobinemia. MetHb is formed by the oxidation of iron from hemoglobin. The oxidation reaction affects the ability of hemoglobin to bind oxygen, leading to tissue hypoxia [78]. A small percentage of MetHb (1–2%) is commonly found in human and animal blood, but does not produce adverse effects. Normal human MetHb levels range from 1.9% to 3.8% [79]. Levels higher than 10–15% (depending on the absolute quantity of available hemoglobin) cause mucosal cyanosis, dyspnea, anxiety, fatigue, confusion, dizziness, tachypnea, and convulsions. Death can occur when the proportion goes over 70–80% [80,81]. The causes are diverse, but nitrites and nitrates are among the most involved substances in the development of this condition.

Due to the generous nitrite and nitrate content of PAW, we expected the percentage of MetHb to vary significantly in mice, given this water daily for 90 days. However, the MetHb levels recorded in the PAW group did not differ significantly from those recorded in the control group (*p* > 0.05). Moreover, the averages of these measurements are not only well below the clinically symptomatic threshold of 10%, but actually around the normal upper limit. No symptoms were observed in mice during the 90 days of the experiment. The presence of an approximately similar MetHb percentage in the control group could be explained by the interaction of chlorides in tap water with hemoglobin [80].

The absorption of ingested nitrate ions is usually achieved in the upper segment of the small intestine, with a bioavailability of 100%, followed by a quick distribution in the body. A probable explanation for this low percentage of MetHb (<4%) in the mice watered with PAW is related to the interactions between reactive nitrogen species (RNS) with the organic material in the digestive tract, which neutralizes them. According to Zhou et al., nitrite can be converted to unstable HNO_2_ under acidic conditions and subsequently decomposed into other nitric oxide species. These reactions may occur at the acidic pH of gastric juice in the forestomach of mice [82].

In dentistry, the use of non-thermal plasma today has several applications: as an antimicrobial in periodontitis, decontamination of dental instruments, in the treatment of mouth ulcers, cancer, to rectify the surface changes of dental implants, to improve the adhesion of materials, in caries treatment and teeth whitening [83,84,85,86]. In a combination treatment with Bioglass^®^, non-thermal plasma can improve the remineralization of enamel lesions [87]. Remineralization and demineralization are processes where various ions are integrated in or removed from the hydroxyapatite crystals of hard tissues (enamel, dentin, cement and bone). Chemical demineralization of teeth is caused by acidic foods and beverages, and by the inflammatory process induced by microbial infections by changing the pH of the oral cavity [88,89]. There are no references in the medical literature about the interaction of PAW with the mineral composition of teeth in long-term administration. In our study, despite the acidity of PAW, there were no changes in the mineral composition of the teeth, with the exception of potassium (Table 1), which was increased in the PAW group. Given the low quantities of this element in the teeth of both groups, a possible explanation for the difference could be the normal dynamics of teeth composition caused by the above mentioned processes of demineralization and remineralization.

Calcium phosphate is essential for bone and tooth formation and we expected it to be affected by the acidic pH (pH 2.78 ± 0.12) of PAW, but the Ca/P ratio (% atoms) did not show significant differences (*p* > 0.05). There were small variations in composition, especially in the PAW group where calcium and phosphorus ions increased by 2.68% and 1.87%, respectively. Small percentage increases in the PAW group were also recorded for other elements: O, F, Na, Mg, Al, K. Remineralization and demineralization of the teeth are continuous, dynamic and reversible processes, present throughout the life of an animal. During their existence, the teeth can lose a substantial number of hydroxyapatite mineral ions from their surface without their integrity being destroyed. If the integrity is destroyed, exposure of hydroxyapatite crystals to an oral environment that promotes remineralization (diet, etc.) may lead to the original remineralization [90]. In case of Ca^2+^ deficiency, a much smaller number of substitutions occur in which calcium (~1%) is replaced by other metal ions, including K^+^, Na^+^ and Mg^2+^. This weakens the resistance of hydroxyapatite, making the dentinal matrix much more vulnerable to acid attack. [91]. Bostanaru et al., suggest that long exposure to PAW (10 min contact/day, for nine days) induces the modification of apatite crystals in non-living teeth (in vitro, in the absence of biological processes) [83]. In our experiment, performed in vivo (in the presence of the biomineralization process) it seems that PAW contributes to increasing the resistance of apatite and implicitly to the resistance of teeth to aggressive factors (acid pH, oral microbiota, unbalanced diet) by increasing calcium phosphates and destroying microorganisms involved in periodontal inflammation. SEM (Figure 1) and AFM (Figure 2) images did not reveal significant differences in the surface morphology of the teeth between the two groups. In the case of the PAW group, we are referring to a pH similar to organic acids in the diet (fruits, etc.), which is not very aggressive on the tooth and which does not cause it to be damaged. Also, the contact time of the teeth with PAW must be taken into account: it is reduced to a few seconds at each watering of the mouse, the saliva of the mouse quickly neutralizing this acidic pH.

In our study, mice treated with PAW for 90 days did not die and showed no adverse effects on the structure and homeostasis of major organs, teeth and the immune system, or other clinical and behavioral changes, especially with regard to food and water consumption, which remained at the same level throughout the experiment. Also, there were no significant differences in animal weight. All these results indicate that PAW appears to be safe for mice and suggest it could also be safe for human long-term use, but this fact needs to be further demonstrated by future clinical studies.

## 4. Materials and Methods

### 4.1. Plasma Device (GlidingArc Reactor) and Production of PAW

The device used in our experiment is based on the GlidArc principle, which has the advantage of adjusting the values of the circuit current based on a special power supply, which works with magnetic dispersion fluxes. PAW preparation requires blowing atmospheric air at high speed (10 m/s), a flow rate of 40 L/min, supplied by an air compressor and injected through a stainless-steel nozzle into the plasma area. Plasma is produced by electric discharge in order to maintain the non-thermal plasma character and to create a turbulent regime at the water surface to have an effective mixing of reactive species in water. The operating conditions of this non-thermal plasma generator were previously described by Ursache et al. and Hnatiuc et al. [36,92].

**Figure 5:**
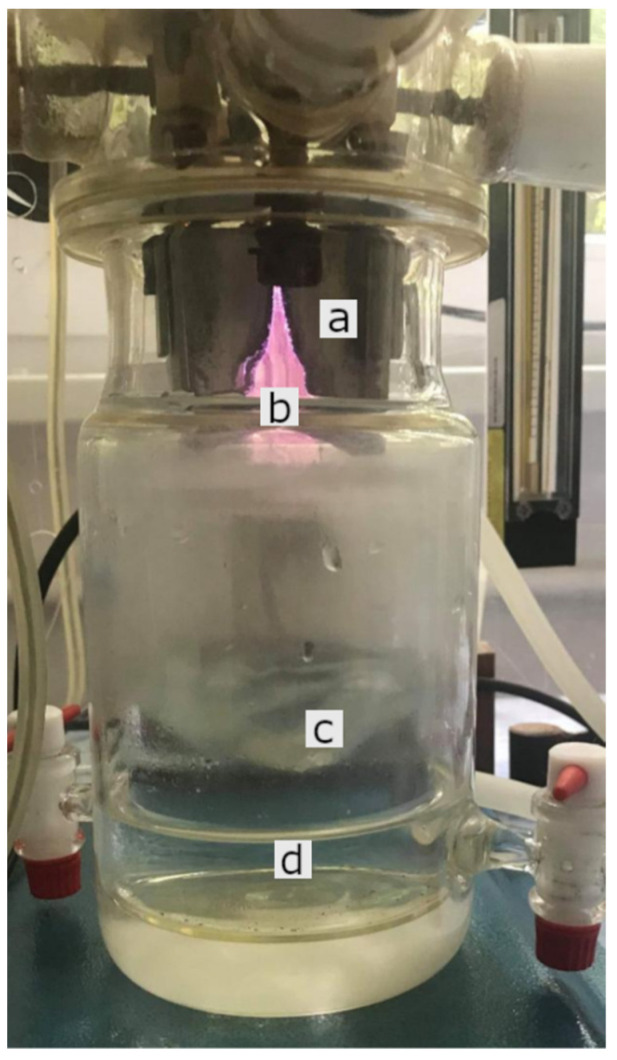
The plasma treatment reactor: (**a**) aluminum electrode, (**b**) plasma discharge, (**c**) plasma-water surface interaction, (**d**) PAW.

PAW was prepared in an electrically insulated cylindrical device made of Pyrex glass (the plasma treatment reactor), where two metal electrodes with divergent profiles were placed (Figure 5); the useful electric discharge occurs between these electrodes. The average value of the power used to prepare the water was calculated at 111.6 W.

A Consort™ C533 multi-parameter analyzer (Consort bvba, Turnhout, Belgium) was used to measure conductivity and pH. The reactive species of ozone (O_3_), hydrogen peroxide, nitrite and nitrate were measured using Spectroquant^®^ analysis kits from Merck (Darmstadt, Germany) [39].

The physico-chemical parameters of the distilled water used in our experiment in the preparation of PAW were as follows: conductivity 5 ± 0.3 μS/cm, pH 6.5 ± 0.16, NO_2_—undetectable, NO_3_—undetectable.

The prepared non-thermal plasma activated water had the following physico-chemical parameters: conductivity 446 ± 25 μS/cm, pH 2.78 ± 0.12, ORP + 1.06 V, NO_2_^–^ 192 ± 10 mg/L, NO_3_^–^ 1550 ± 95 mg/L, H_2_O_2_ 2.6 ± 0.12 mg/L, O_3_ 1.08 ± 0.07 mg/L.

### 4.2. Animals

In our study 20 outbred mice of the CD1 strain purchased two weeks before the start of the experiment from the Cantacuzino Institute in Bucharest were used. The mice were 10-week-old nulliparous females, with a mean weight of 28.85 ± 5.12 g at the beginning of the experiment. Mice acclimation was done under identical conditions of temperature (22 ± 0.7 °C) and humidity (50 ± 10%), with a light/dark cycle of 12 h. Each experimental group was housed in autoclavable polycarbonate cages of 1500 cm^2^, approximately 300 cm^2^/mouse. The animals had a permanent access to water (ad libitum) (autoclavable bottles with drip system) and standardized food—Cantacuzino Institute—with the following composition: 23% protein, 10% fat, 50% carbohydrates, 8% crude fiber and 9% vitamin-mineral premix, calcium carbonate and phosphate, amino acids.

### 4.3. Experimental Design

The experiment was performed on 20 CD1 mice divided into two groups: the control group watered with tap water (*n* = 10), and the experimental group (PAW), watered with PAW (*n* = 10). The experiment took place over 90 days, during which time tap water and PAW were replaced daily at the same time. Thus, a volume of 300 mL of distilled water was treated every morning at 8 o’clock for 10 min, after which PAW was passed into borosilicate bottles used for watering the animals in the experimental group. Our attempt to use PAW in usual plastic (PET) bottles for watering resulted in a failed experiment; PAW interacted with the plastic and led to the release of large amounts of bisphenol with pathological implications on the animals’ health [93].

In order to guarantee that the mice immediately consumed PAW in large quantities, their watering was done only for 12 h, after which they were deprived of water until the next morning, when the whole process was resumed with freshly prepared PAW. This strategy was used to minimize the influence of PAW aging (the reduction of RONS concentration over time).

The two experimental groups were monitored daily, in the same time interval, for 90 days, clinically following: signs of general toxicity such as color changes of the skin or mucous membranes, piloerection, diarrhea, more than 10% weight loss, and behavioral changes—reduced food intake, depression (reduced locomotor activity), abnormal body posture, according to the guidelines of the Organisation for Economic Co-operation and Development (OECD) for testing chemicals [94]. Body weight (absolute and relative) were measured according to Marxfeld et al. [95].

### 4.4. Scanning Electronic Microscopy (SEM), Energy Dispersive X-ray Analysis (EDX) and Atomic Force Microscopy (AFM)

**Figure 6:**
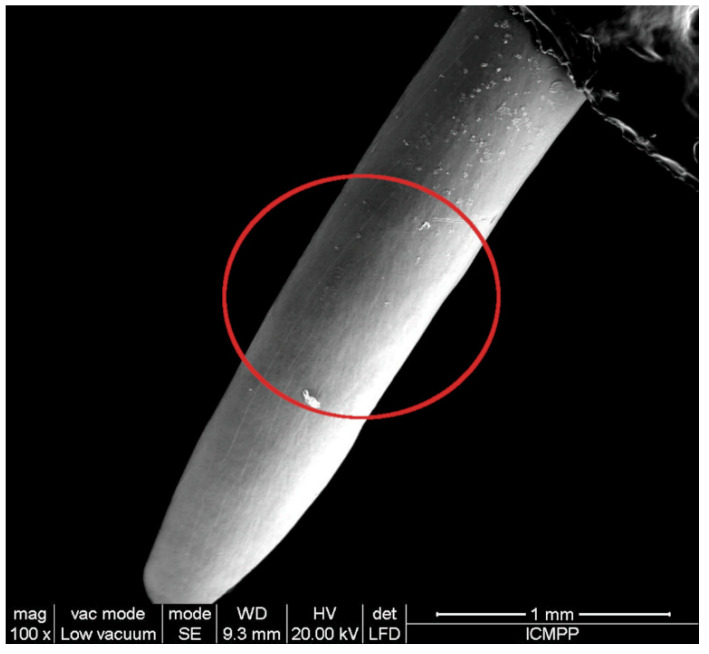
Lingual surface of CD1 mouse incisive (SEM). The red circle shows the area from which EDX and AFM data were collected.

The micro-morphology of mice teeth was investigated with a Quanta 200 scanning electron microscope (SEM, FEI Company, Czech Republic), working at 20 kV in low vacuum mode, with a large field detector (LFD). The mice teeth were placed on aluminum stubs with double-sided adhesive carbon tape and were analyzed without any coating in order to preserve the morphology intact. For the energy-dispersive X-ray analysis (EDX), the microscope was equipped with a specific detector and the compositional characterization was evaluated with the corresponding EDAX Genesis software (Ametek, Berwyn, PA, USA). The analyzed area was in the middle third of the lingual surface of the teeth (Figure 6).

Atomic force microscopy (AFM) was also used to study the influence of PAW on the surface of mice teeth. The surface morphology was investigated using an Ntegra Spectra Atomic Force Microscope (NT-MDT, Moscow, Russia). Silicon cantilever tips (NSG 10, NT-MDT, Moscow, Russia) with gold reflecting coating, a resonance frequency of 140–390 kHz, a force constant of 3.1–37.6 N m^−1^ and a tip curvature radius of 10 nm were used. The surface topographies were obtained in tapping mode, in order to minimize wear and tear of the teeth surface and under ambient conditions. The AFM images were recorded over an area of 10 μm × 10 μm in the middle third of the lingual surface of the teeth with an acquisition speed of 13 μm/s and a lateral resolution of 512 × 512 pixels. A first-order plane was fitted to the AFM sensheight images and subtracted to correct for sample tilt. No special sample preparation for AFM measurements was needed. AFM image analysis was performed with Gwyddion software version 2.58 (http://gwyddion.net/ accessed on 20 February 2021) and surface roughness Sq (root-mean-square roughness) was determined based on the surface height images.

### 4.5. Histopathology and Immunohistochemistry

Immediately after euthanasia with isoflurane 2%, tissue samples (tongue, oral mucosae, gastric mucosae, duodenum, jejunum, colon, liver, pancreas and sublingual glands, myocardium, coronary artery, lung, cortex, cerebellum, kidney, adrenal glands, spleen and lymph node) were harvested from both the control and the experimental group, and fixed in 10% buffer formalin, embedded in paraffin and Trichrome Masson Stained (TMS). In addition to the histopathological examination, which searched for preneoplastic and neoplastic changes of the lingual, esophageal and gastric epithelium (dysplasia, metaplasia, anisocytosis, anisocariosis, nuclear hypertrophy, N/C rapport, and chromatin condensation), immunohistochemistry was performed for two cell proliferation markers, PCNA (proliferating cell nuclear antigen) and KI-67, in order to estimate the proliferation grade of epithelial cell populations [96,97,98].

An immunohistochemical staining for Ki-67 and PCNA was performed to improve the diagnostic accuracy in a malignant cell proliferation (proliferative potential and for identifying the proliferation status of tumor tissue), along with a histological assessment for possible tumors of these epitheliums, as a possible effect of PAW long-term administration.

The immunohistochemical stain for Ki-67 was performed using mouse monoclonal antibodies, from Leica-Novocastra (Leica Biosystems, Wetzlar, Germany). Bond RX immunostainer (Leica Biosystems, Wetzlar, Germany) was used for immunohistochemical staining. The sections were de-paraffinized and sequentially treated for antigen epitope retrieval (retrieval solution, pH 6) and endogenous peroxidase blocking, following an overnight incubation with primary antibodies (1/200 dilution). Following a 5-min application of DAB chromogen solution, the section was counterstained with Mayer hematoxylin (Dako, Santa Clara, CA, USA). This was followed by dehydration and mounting for microscopic examination.

To validate the staining procedure, a positive control slide containing tissue section from tonsil with known diffuse immunoreactivity for Ki-67 was used. For a negative control, we used the buffer solution rather than the primary antibody.

The immunohistochemical stain for PCNA was performed using mouse monoclonal antibodies—ready to use, from ThermoFisher Scientific (Waltham, MA, USA). The sections were de-paraffinized, no retrieval needed, following an overnight incubation with primary antibodies (ready to use). Using the Ultravision Quanto Detection System-HRP DAB (Thermofisher Scientific, Waltham, MA, USA), the section was counterstained with Mayer hematoxylin (Dako, Santa Clara, CA, USA). This was followed by dehydration and mounting for microscopic examination.

The expression of Ki-67and PCNA was assessed based on the distribution and proportion of marked epithelium cells.

### 4.6. Hematology

Blood collection for hematological investigations was performed in sterile Kima^®^ 500 µL, K3 EDTA vacuum tubes (Arzergrande, Italy) kept at room temperature. To prevent coagulation, homogenization of the blood with the anticoagulant in the tube was performed by gently rotating the tube vertically 5–6 times. Harvesting was performed at the end of the experiment, by cardiac puncture and under isoflurane anesthesia. Hematological determinations were performed with the ABAXIS VetScan^®^ HM2 Hematology System (Zoetis UK Ltd, Leatherhead, UK), for the following parameters: white blood cells (WBC), lymphocytes (LYM), monocytes (MON), neutrophils (NEU), eosinophils (EOS), basophils (BAS), red blood cells (RBC), hemoglobin (HGB), hematocrit (HCT), mean corpuscular volume (MCV), mean corpuscular hemoglobin (MCH), mean corpuscular hemoglobin concentration (MCHC), red cell distribution width (RDWs), red cell distribution width % (RDWc), platelets (PLT), plateletcrit (PCT), mean platelet volume (MPV), platelet distribution width (PDWs) and platelet distribution width % (PDWc).

### 4.7. Blood Biochemistry

Blood collection for biochemical investigations was performed in 500 µL vacuum tubes with heparin that were kept at room temperature; the tubes were gently vertically homogenized 5–6 times to prevent coagulation. The determination of the biochemical parameters was performed with the help of the IDEXX VetTest Chemistry Analyzer (Westbrook, ME, USA), a device that uses the “dry-slide” technique that ensures the filtration of interference substances from the sample, thus being able to analyze hemolyzed samples as well. The analyzed parameters were: alkaline phosphatase (ALKP), alanine aminotransferase (ALT), blood urea nitrogen (BUN), creatinine (CREAT), glucose (GLU), and total proteins (T-PRO).

### 4.8. Immunological Examination

Blood was collected in 500 µL vacuum tubes without anticoagulant and with separating gel (Vacutest Kima, activating clot). After collection, the blood sample was allowed to stand at room temperature for 30–60 min, after which it was centrifuged at 5000 rpm for 5 min, separating the serum. The detection of cytokines in the serum was performed by multiplex ELISA (Bio-Rad, Hercules, CA, USA) and data interpretation was performed using the acquisition system Bio-Plex Manager™ MP Software—Bio-Plex Data Pro™ Software 1.2. The following cytokines were measured: interleukins (IL-1a, IL-1β, IL-2, IL-3, IL-4, IL-5, IL-9, IL-10, IL-12 (p40), IL-12 (p70), IL-13, IL-17A, IL-17F, IL-21, IL-22, IL-23 (p19), IL-25, IL-27, IL-31, IL-33), EOTAXIN, granulocyte colony-stimulating factor (G-CSF), granulocyte-macrophage colony-stimulating factor (GM-CSF), interferon gamma (IFN-γ), keratinocyte chemoattractant (KC), CD40 ligand (CD40L), macrophage inflammatory protein-3 alpha (MIP-3α), monocyte chemoattractant protein 1 (MCP-1), macrophage inflammatory protein 1 alpha and beta (MIP-1α and MIP-1β), chemokine ligand 5 (RANTES), and tumor necrosis factor alpha (TNF-α).

### 4.9. Determination of Methemoglobin (MetHb)

The study was performed on 10 CD1 female mice, divided into two groups, the control group (*n* = 5) watered with tap water and the experimental group (*n* = 5) watered ad libitum with PAW, daily for 90 days. At the end of the experiment, each mouse had 200 µL of blood collected by puncturing the submaxillary vein. The measurement of MetHb level was done in relation to oxyhemoglobin, by spectrophotometric absorption at 630 nm (MetHb) and 540 nm (oxyhemoglobin), hemolyzed and stabilized in phosphate buffer M/60, where both pigments coexist.

To determine MetHb, 100 μL of whole blood was mixed with 100 μL of 1% saponin, and after homogenization and hemolysis of the blood, 6 mL of M/60 phosphate buffer with pH 6.8 was added to the mixture. In the case of oxyhemoglobin, a tube with 3 mL of M/60 phosphate buffer with pH 6.8 was used, in which 300 µL of mixture was added from the first tube (the one for MetHb), being then very well homogenized.

The absorbance measurement was performed with a Boeco UV—VIS spectrophotometer (Hamburg, Germany) at 630 nm for MetHb (tube A) and at 540 nm for oxyhemoglobin (tube B). The percentage of MetHb (% MetHb) was calculated according to the formula:

% MetHb = absorbance of tube A × 100/(absorbance of tube A + absorbance of tube B × 10).

All measurements were performed on a control containing M/60 phosphate buffer with pH 6.8 [79]. If the MetHb level was below 10%, it was considered safe for mouse and human health.

### 4.10. Statistical Analysis

The differences between the control group and the PAW group were assessed with the unpaired t test, which was performed using GraphPad Prism version 9.2.0 for Windows, GraphPad Software, San Diego, CA, USA, www.graphpad.com (accessed on 9 September 2021). Two-tailed *p* values were calculated and the differences were considered statistically significant when *p* < 0.05.

### 4.11. Ethical Implications

The study was conducted in accordance with the 2019 consolidated version of the Directive 2010/63/EU legal regulations on the protection of animals used for scientific purposes.

## 5. Conclusions

This study provides the latest and most complete information on the toxicity of long-term consumption of plasma activated-water (PAW) in CD1 mice.

Long-term consumption of PAW has no negative effect on CD1 mice body homeostasis.

Long-term PAW consumption did not appear to significantly alter the chemical composition of the teeth.

Our results showed that PAW does not induce functional and histological changes in the liver, kidneys, heart, spleen, brain, endocrine system (adrenal glands, pancreas) and digestive system. Moreover, it does not cause changes in hematological and biochemical blood parameters, which characterize the body’s homeostasis. Also, it did not cause an inflammatory status, its absence being highlighted both histologically and by the fact that there are no significant changes of the proinflammatory status (cytokines) in the two groups.

Long-term PAW consumption appears to be safe for CD1 mice.

Future studies will focus on the interaction of Paw with the mouse microbiome and the influence of this interaction on health in the case of long-term consumption of PAW.

## Figures and Tables

**Table 1:** Teeth composition—% atoms.

Minerals	Control(Mean ± SD)	PAW(Mean ± SD)
Carbon (C)	66.19 ± 6.04	58.39 ± 7.85
Nitrogen (N)	10.28 ± 1.69	8.37 ± 1.41
Oxygen (O)	19.70 ± 4.34	24.13 ± 5.10
Fluorine (F)	0.14 ± 0.06	0.20 ± 0.16
Sodium (Na)	0.11 ± 0.07	0.18 ± 0.06
Magnesium (Mg)	0.16 ± 0.11	0.29 ± 0.15
Aluminum (Al)	0.05 ± 0.03	0.12 ± 0.07
Silicon (Si)	0.05 ± 0.02	0.05 ± 0.01
Phosphorus (P)	1.52 ± 1.18	3.39 ± 1.59
Chlorine (Cl)	0.00 ± 0.00	0.01 ± 0.01
* Potassium (K)	0.02 ± 0.01	0.06 ± 0.02
Calcium (Ca)	2.07 ± 1.57	4.75 ± 2.19

The asterisk (*) marks statistically significant differences.

**Table 2:** The absolute and relative weight of the essential organs.

ORGAN	Control (Mean ± SD)		PAW (Mean ± SD)	
	Absolute (g)	Relative (%)	Absolute (g)	Relative (%)
Heart	0.18 ± 0.02	0.49 ± 0.04	0.17 ± 0.03	0.49 ± 0.08
* Spleen	0.16 ± 0.03	0.43 ± 0.04	0.13 ± 0.02	0.36 ± 0.03
Liver	1.45 ± 0.01	3.96 ± 0.37	1.33 ± 0.14	3.87 ± 0.31
Kidneys	0.45 ± 0.08	1.22 ± 0.12	0.39 ± 0.05	1.14 ± 0.10
Brain	0.49 ± 0.01	1.34 ± 0.15	0.50 ± 0.04	1.43 ± 0.11
Bodyweight	36.81 ± 4.95		34.43 ± 3.15	

The asterisk (*) marks statistically significant differences.

**Table 3:** Hematological parameters.

Parameter	Control	PAW
RBC (10^12^/L)	9.39 ± 0.55	9.22 ± 1.20
HGB (g/dL)	14.12 ± 0.93	13.05 ± 2.00
HCT (%)	45.48 ± 3.66	44.19 ± 7.26
MCV (fl)	48.00 ± 3.33	47.80 ± 4.96
MCH (pg)	14.64 ± 0.83	14.28 ± 0.91
MCHC (g/dL)	31.84 ± 2.62	31.64 ± 2.40
RDWc (%)	18.90 ± 1.55	18.81 ± 1.19
RDWs (%)	35.56 ± 6.73	33.83 ± 7.48
PLT (10^9^/L)	367.10 ± 147.10	368.70 ± 118.10
PCT (%)	0.35 ± 0.05	0.32 ± 0.93
MPV (fl)	6.47 ± 0.26	6.66 ± 0.42
PDWc (%)	28.30 ± 1.45	29.10 ± 2.11
PDWs (fl)	7.84 ± 0.79	7.84 ± 0.87
WBC (10^9^/L)	3.59 ± 0.82	3.77 ± 1.37
NEU (10^9^/L)	0.36 ± 0.07	0.34 ± 0.10
LIMF (10^9^/L)	2.94 ± 0.76	3.32 ± 1.22
MONO (10^9^/L)	0.11 ± 0.02	0.12 ± 0.05
EOS (10^9^/L)	<min	<min
BASO (10^9^/L)	<min	<min
NE (%)	11.94 ± 2.84	10.80 ± 1.88
LIMF (%)	84.61 ± 3.76	85.12 ± 2.87
MONO (%)	3.60 ± 0.92	3.27 ± 0.99
EOS (%)	-	-
BASO (%)	-	-

<min = below the lower limit of detection.

**Table 4:** Blood biochemical parameters.

Parameter	Control	PAW
BUN (mg/dL)	27.20 ± 5.59	29.90 ± 6.36
GLU (mg/dL)	137.50 ± 30.28	142.90 ± 11.97
ALP/ALK (U/L)	126.60 ± 20.44	135.10 ± 15.41
GPT/ALT (U/L)	21.10 ± 8.27	20.40 ± 4.97
T-PRO (g/dL)	5.25 ± 0.46	5.15 ± 0.69
CREAT (mg/dL)	0.96 ± 0.18	0.94 ± 0.20

**Table 5:** Cytokine levels in CD1 mice.

Cytokine	Control	PAW
IL-1a	4.17 ± 0.71	3.54 ± 0.37
IL-1b	<min	<min
IL-2	3.20 ± 0.01	2.67 ± 5.98
IL-3	3.04 ± 0.61	2.17 ± 0.46
IL-4	0.78 ± 0.49	<min
IL-5	3.68 ± 0.76	1.92 ± 1.19
IL-6	2.94 ± 0.26	2.30 ± 0.39
IL-9	13.45 ± 2.5	6.89 ± 6.33
IL-10	44.40 ± 27.65	15.25 ± 4.91
IL-12 (p40)	274.06 ± 46.64	214.86 ± 18.95
IL-12 (p70)	82.91 ± 13.87	60.61 ± 7.65
IL-13	35.62 ± 10.56	60.61 ± 30.20
IL-17A	51.32 ± 16.76	12.78 ± 16.19
IL-17F	0.36 ± 0.32	0.29 ± 0.32
IL-21	<min	<min
IL-22	1.29 ± 0.64	1.71 ± 0.52
IL-23 (p19)	4.10 ± 4.30	2.55 ± 2.70
IL-25	2.59 ± 1.53	0.53 ± 0.26.
IL-27	1.48 ± 1.46	0.34 ± 0.19
IL-31	3.47 ± 4.75	3.43 ± 4.69
IL-33	<min	<min
Eotaxin	249.84 ± 69.02	211.09 ± 73.41
G-CSF	44.21 ± 7.69	38.29 ± 13.06
GM-CSF	24.10 ± 2.72	19.91 ± 4.35
IFN-γ	11.03 ± 1.88	5.72 ± 2.02
KC	14.15 ± 1.53	21.39 ± 23.43
CD40L	<min	<min
MIP-3α	0.60 ± 0.40	0.87 ± 1.02
MCP-1	85.80 ± 15.36	52.14 ± 13.91
MIP-1α	2.47 ± 0.50	1.10 ± 0.70
MIP-1β	6.42 ± 4.25	6.72 ± 5.09
RANTES	26.08 ± 5.76	17.64 ± 2.07
TNF-α	45.54 ± 10.67	43.07 ± 14.74

<min = below the lower limit of detection.

## Data Availability

Our own data presented in this study are available on request from the corresponding author.

## Supplementary Materials

The following are available online at https://www.mdpi.com/article/10.3390/ijms222111534/s1.
