# Supplementary figures and images for: Toxicity Assessment of Long-Term Exposure to Non-Thermal Plasma Activated Water in Mice
Source: Int J Mol Sci. 2021 Oct 26;22(21):11534. doi: 10.3390/ijms222111534 (PMC8583710; doi:10.3390/ijms222111534)

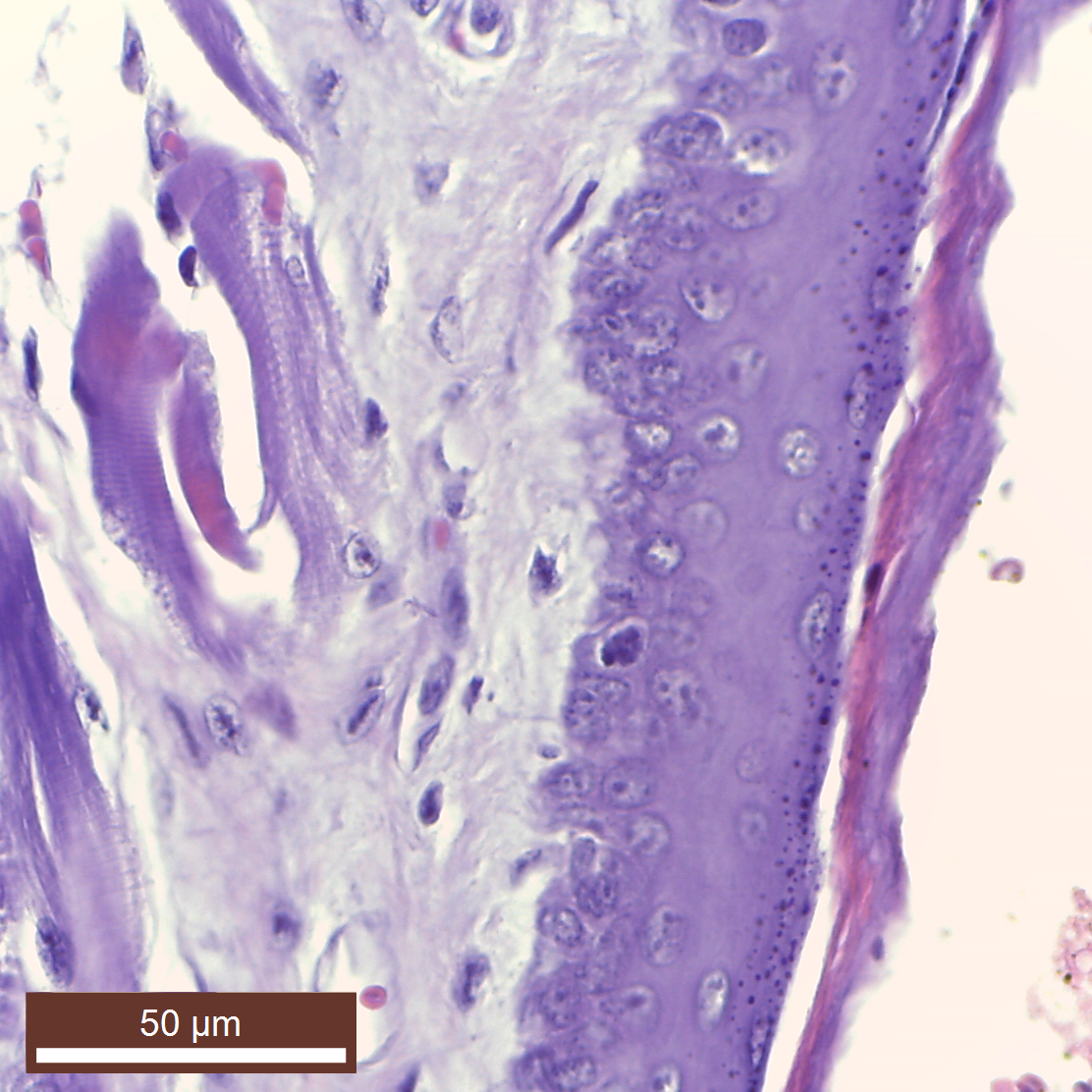

Supplement: Supplementary file 1 [file ijms-22-11534-s001.zip › Figure-S1-oral-mucosa.tif]

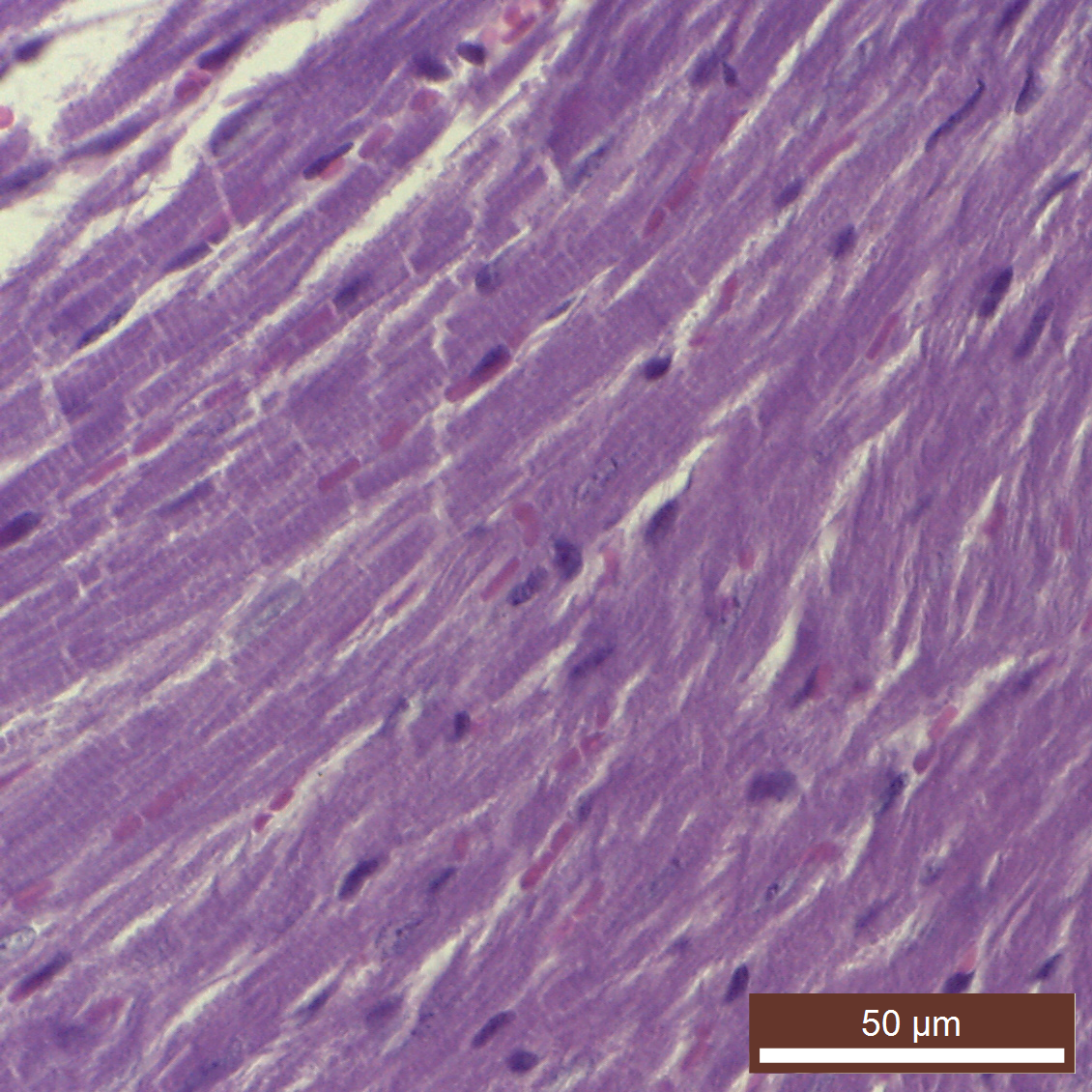

Supplement: Supplementary file 1 [file ijms-22-11534-s001.zip › Figure-S10-myocardium.tif]

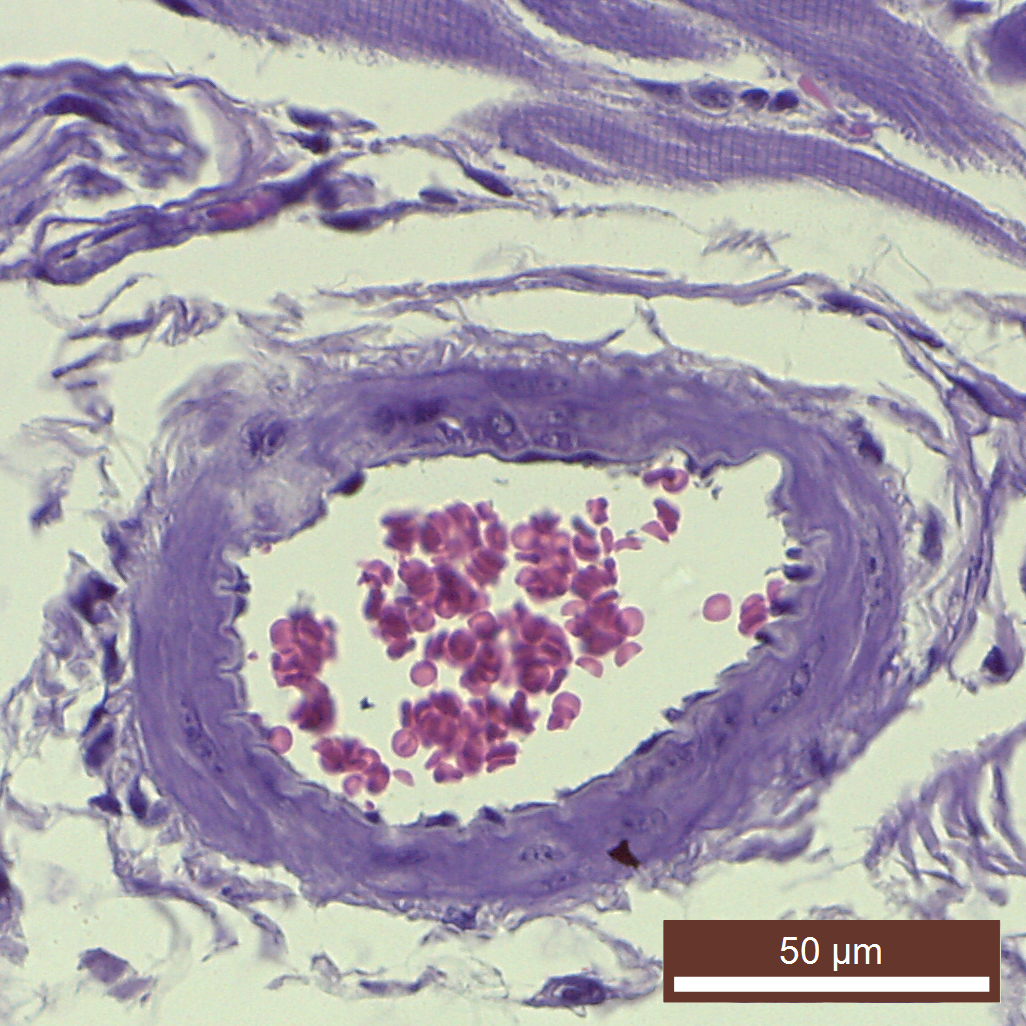

Supplement: Supplementary file 1 [file ijms-22-11534-s001.zip › Figure-S11-coronary-artery-branch.tif]

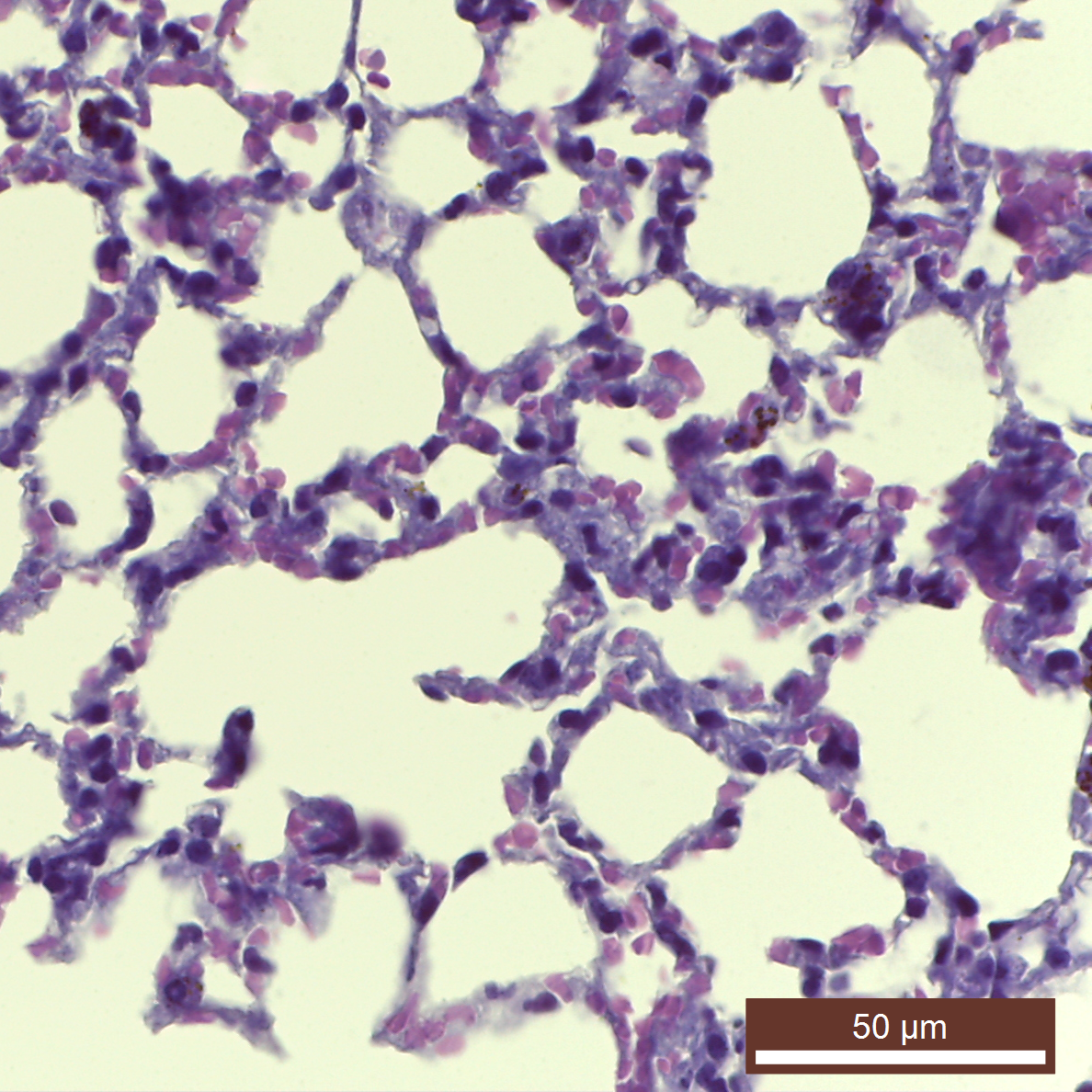

Supplement: Supplementary file 1 [file ijms-22-11534-s001.zip › Figure-S12-lung.tif]

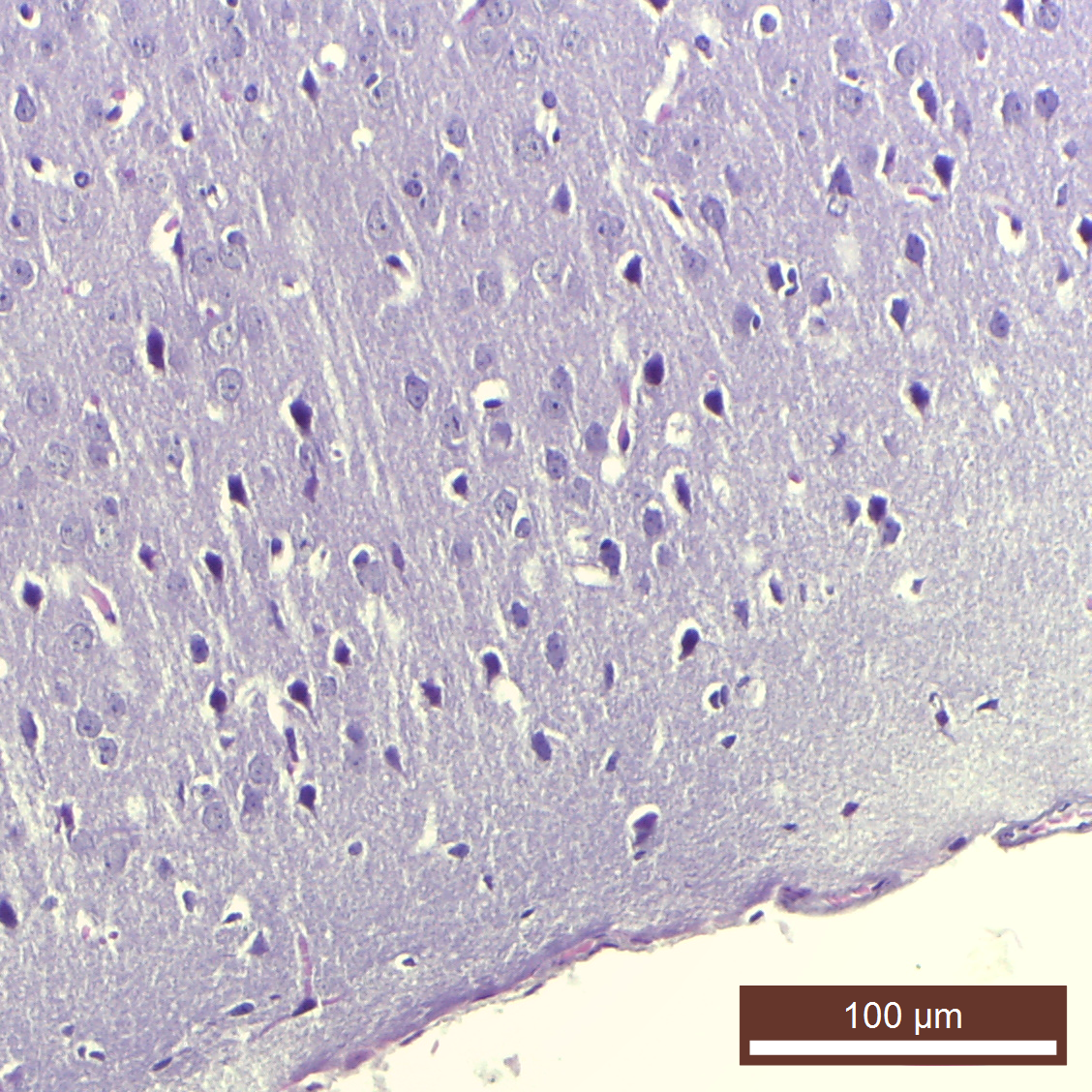

Supplement: Supplementary file 1 [file ijms-22-11534-s001.zip › Figure-S13-cerebral-cortex.tif]

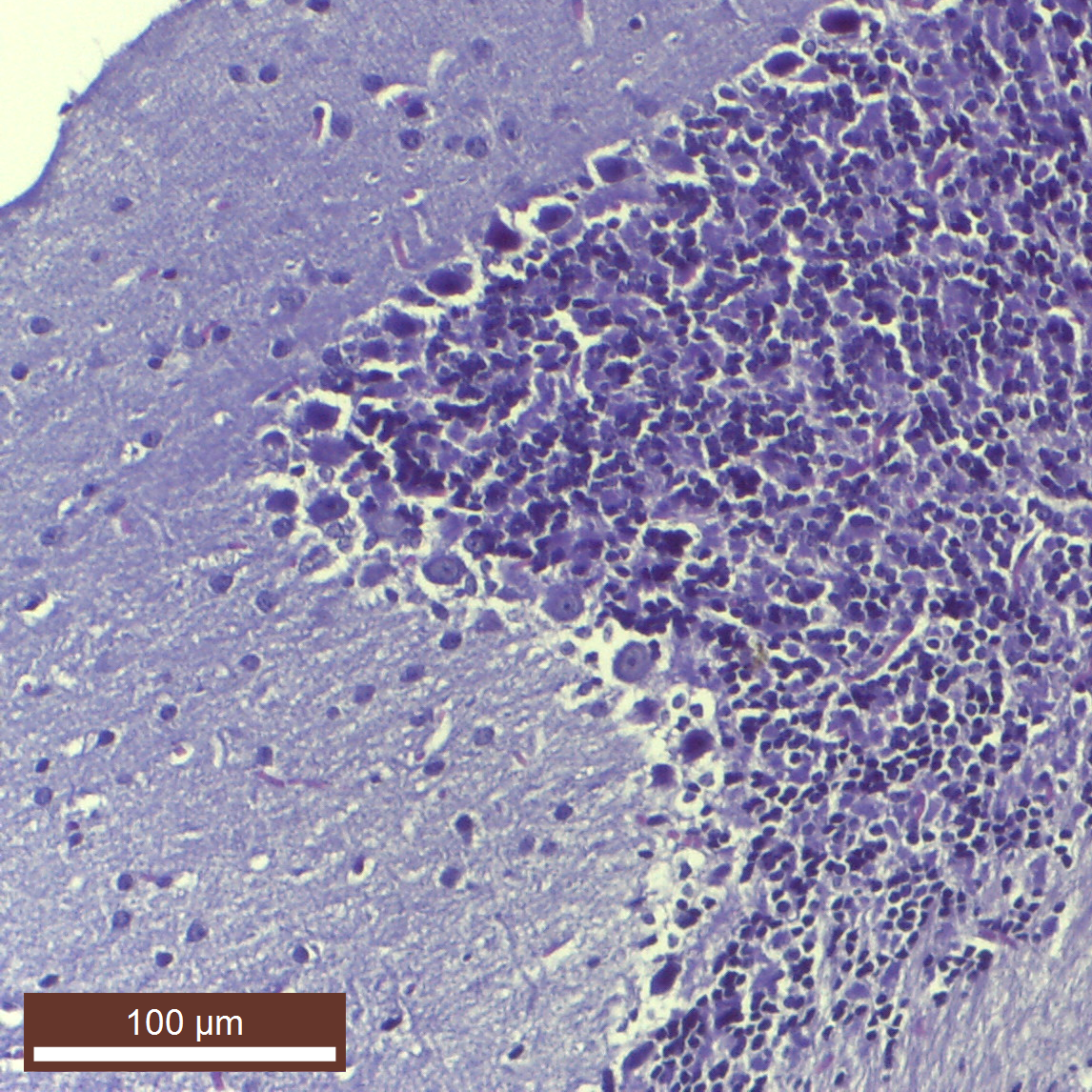

Supplement: Supplementary file 1 [file ijms-22-11534-s001.zip › Figure-S14-cerebellum.tif]

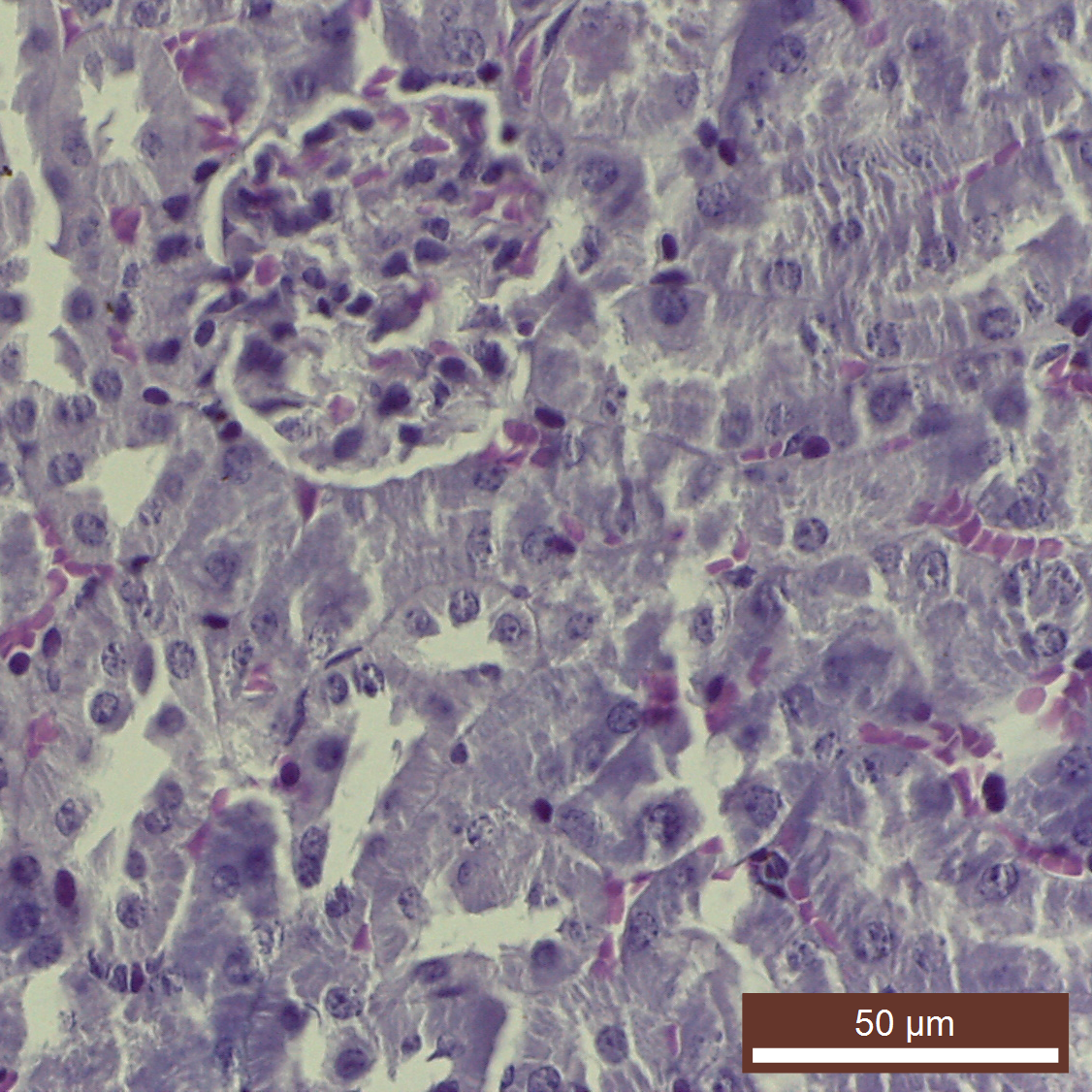

Supplement: Supplementary file 1 [file ijms-22-11534-s001.zip › Figure-S15-kidney-cortex.tif]

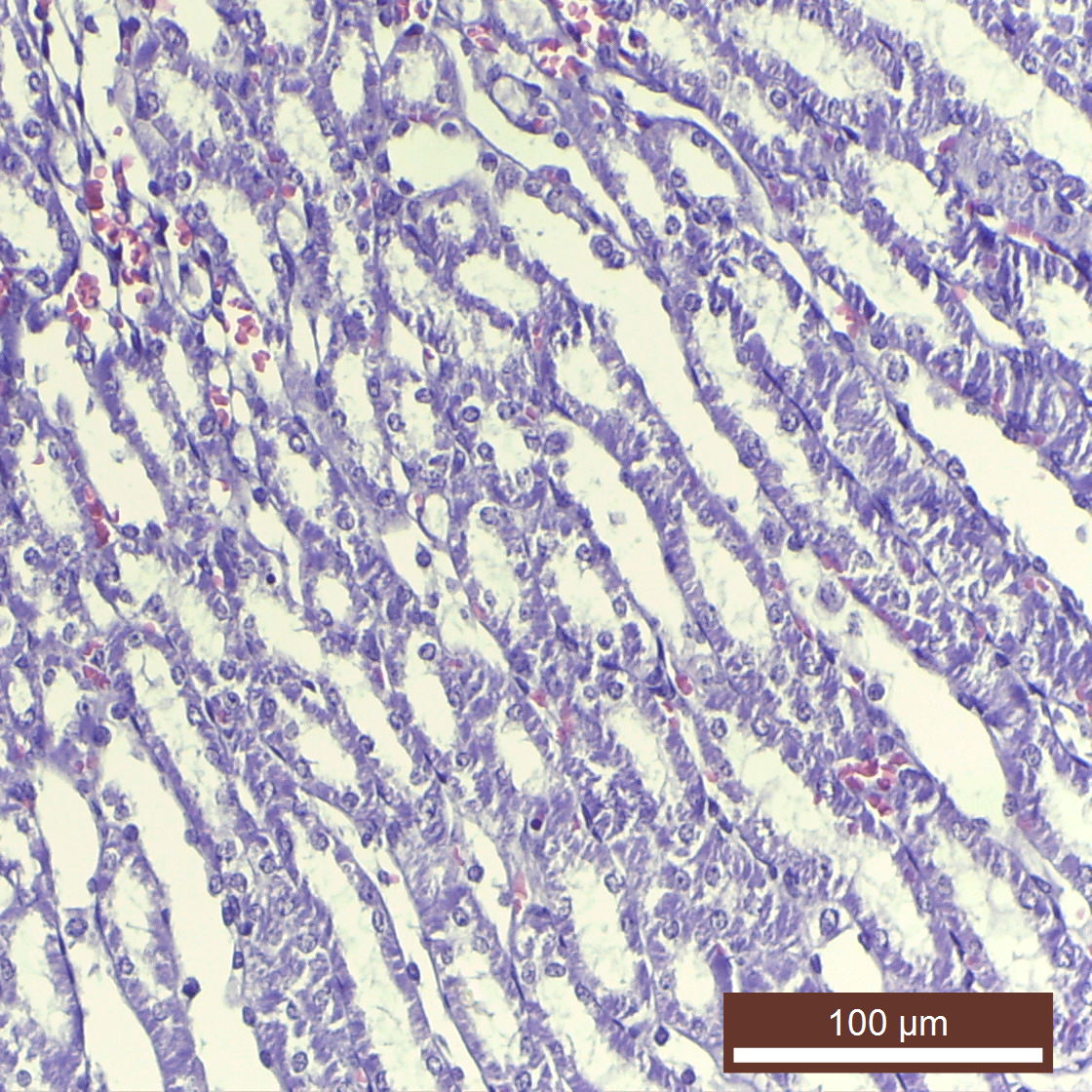

Supplement: Supplementary file 1 [file ijms-22-11534-s001.zip › Figure-S16-kidney-medulla.tif]

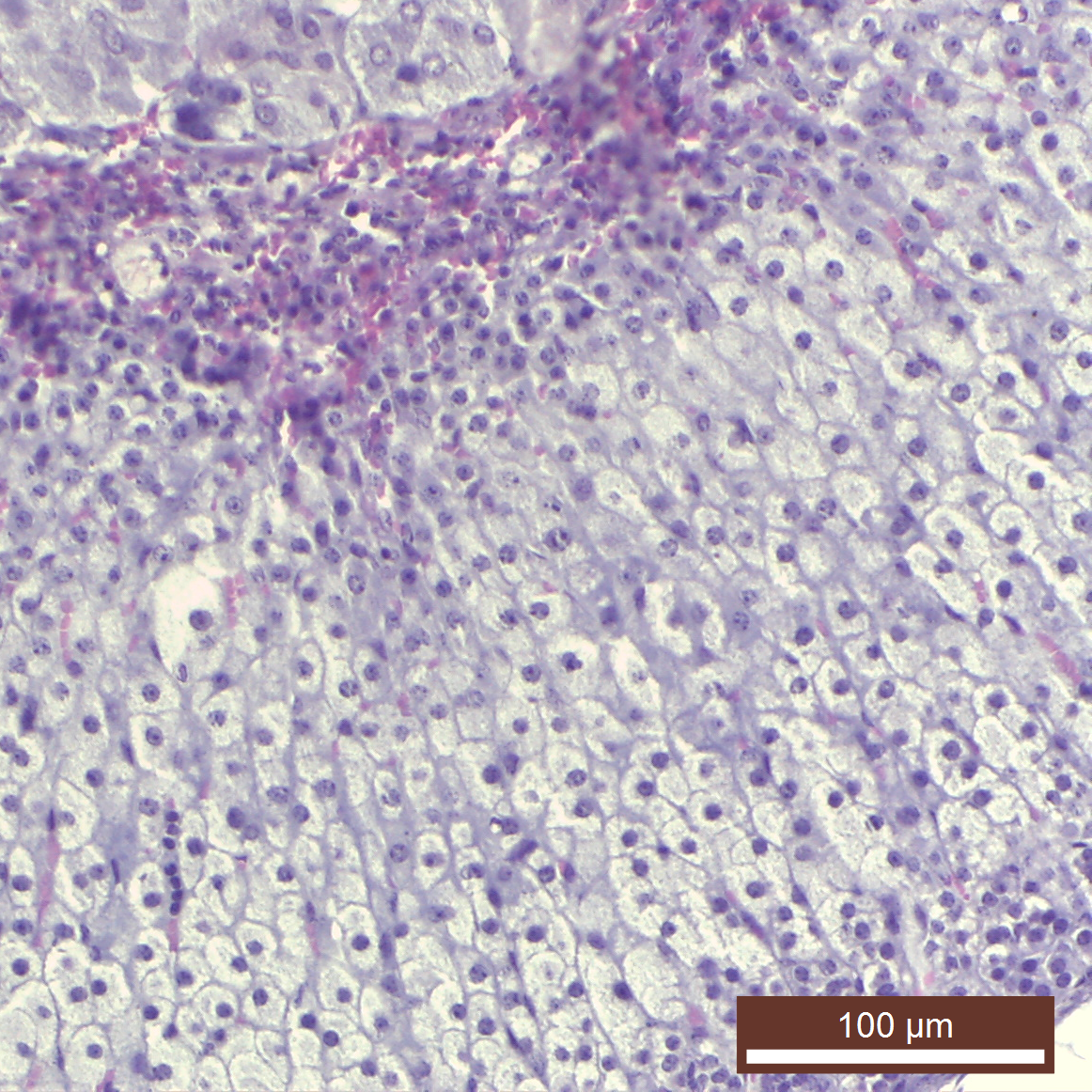

Supplement: Supplementary file 1 [file ijms-22-11534-s001.zip › Figure-S17-adrenal-gland-cortex.tif]

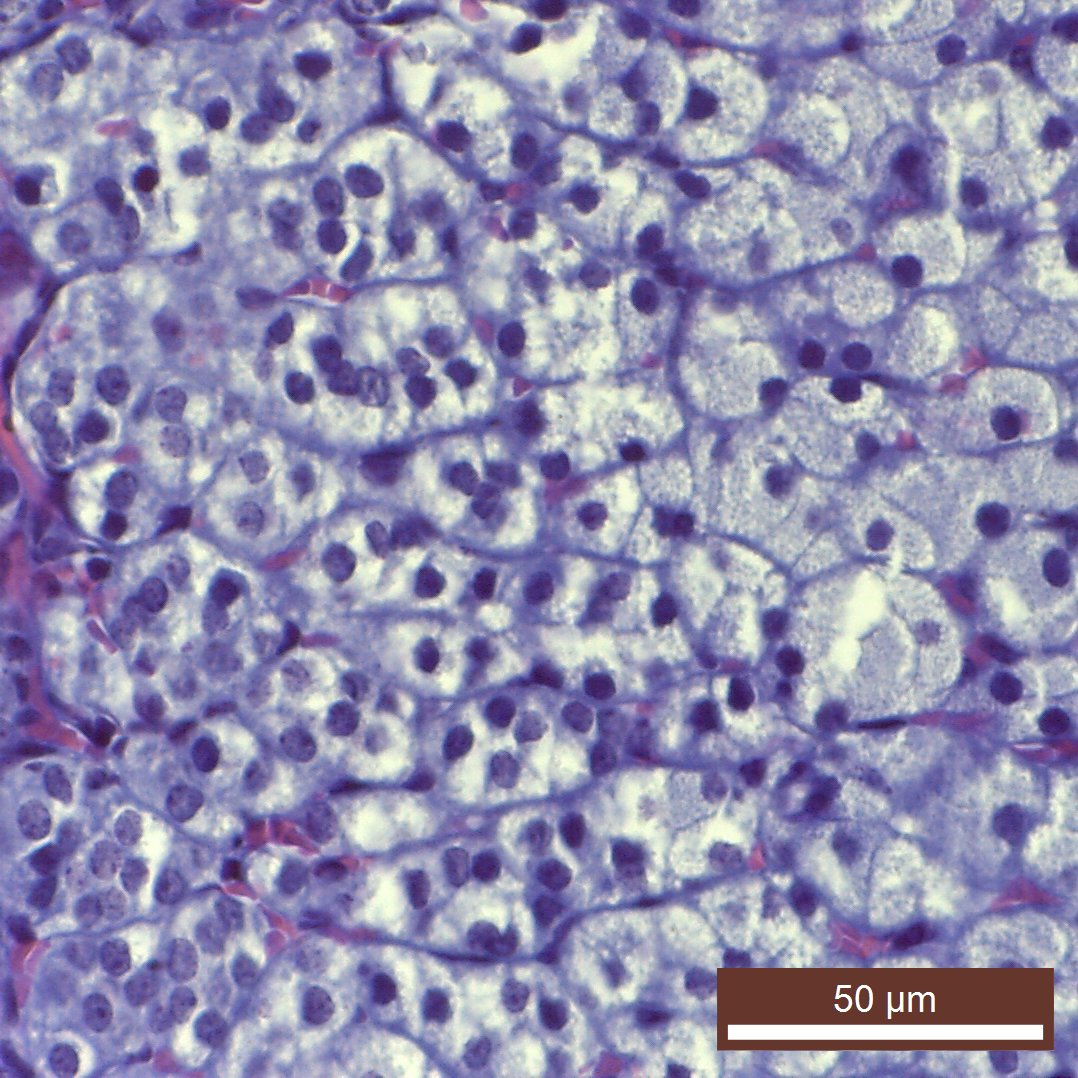

Supplement: Supplementary file 1 [file ijms-22-11534-s001.zip › Figure-S18-adrenal-gland-medulla.tif]

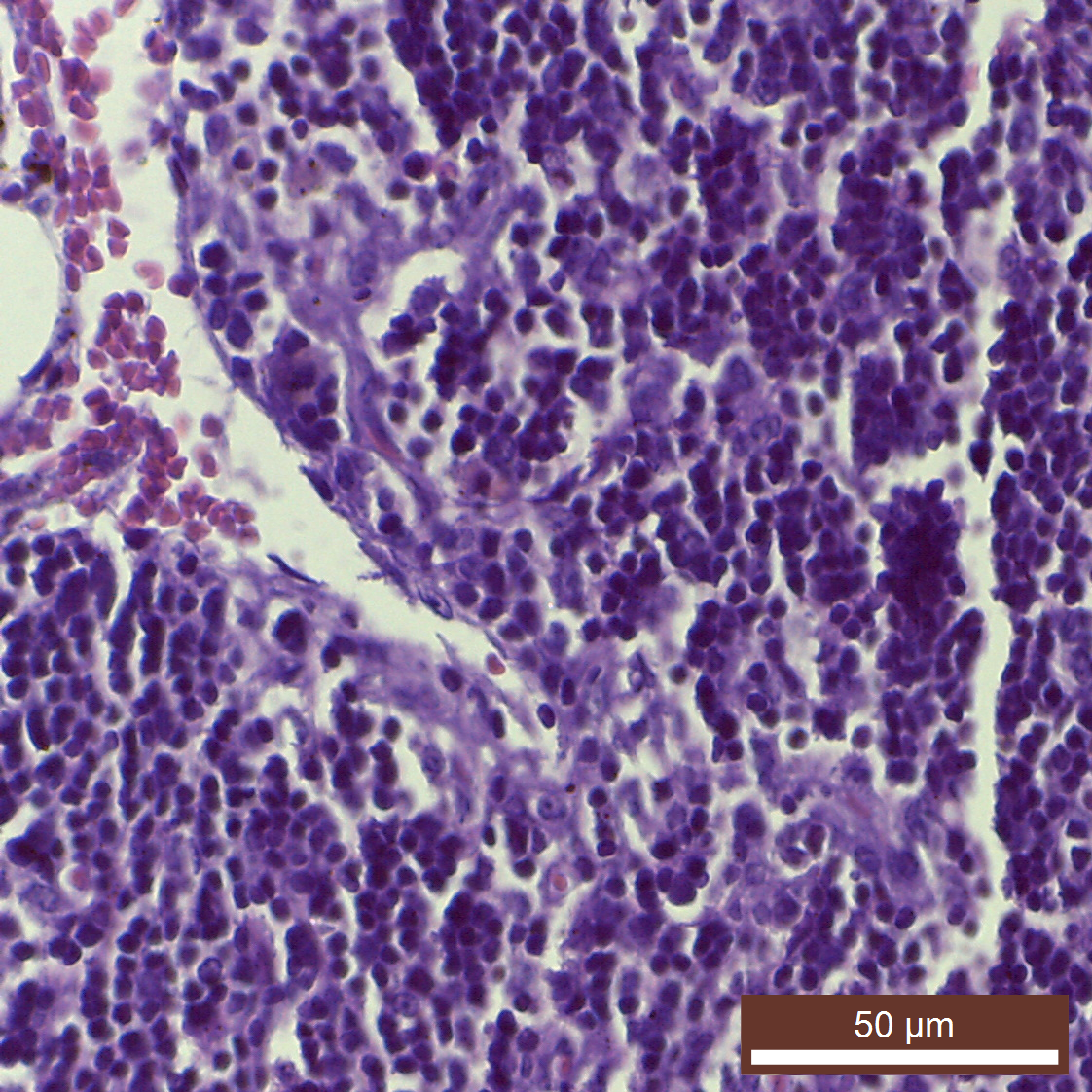

Supplement: Supplementary file 1 [file ijms-22-11534-s001.zip › Figure-S19-epigastric-lymph-node.tif]

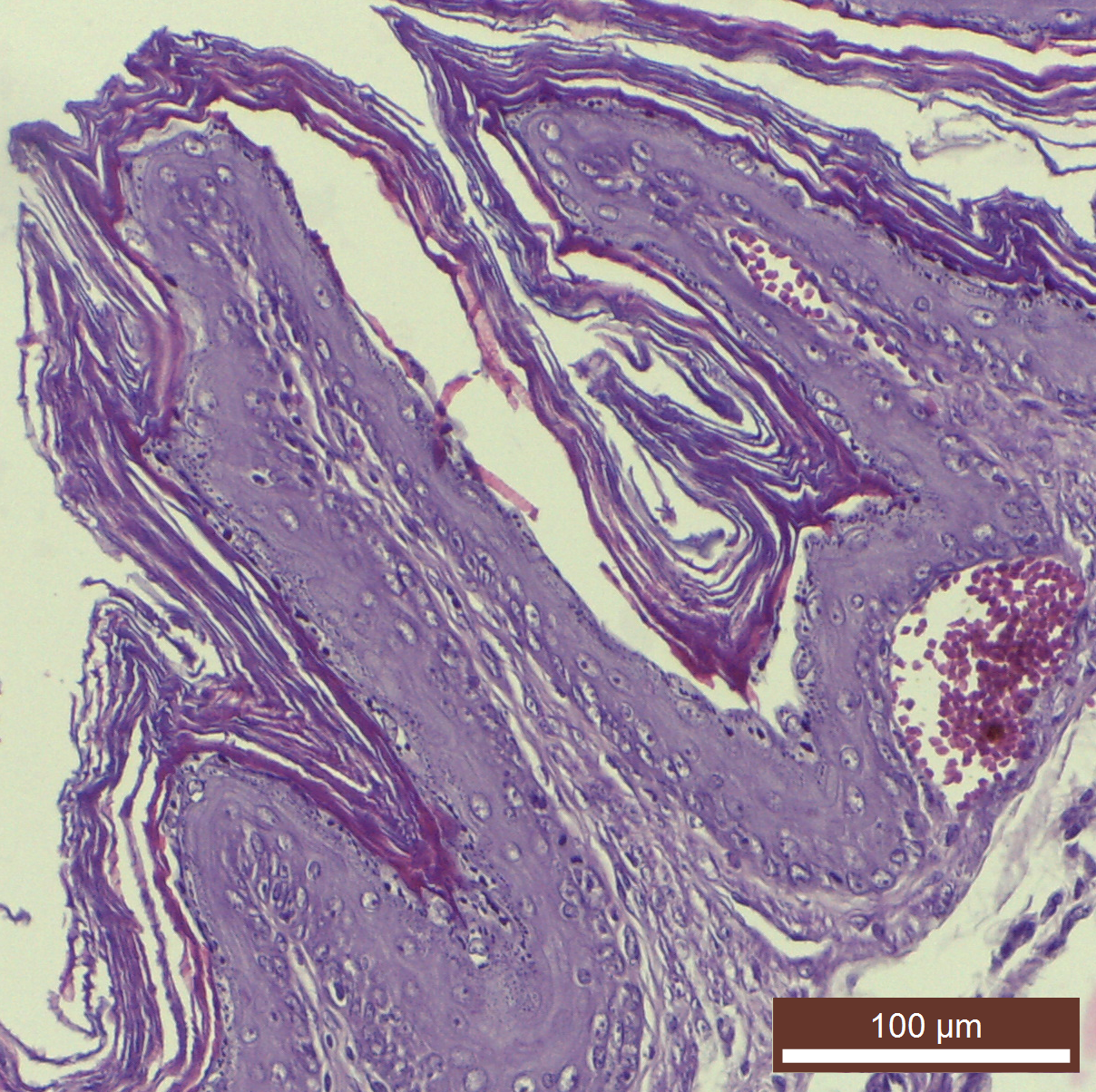

Supplement: Supplementary file 1 [file ijms-22-11534-s001.zip › Figure-S2-gastric-mucosa-keratinized.tif]

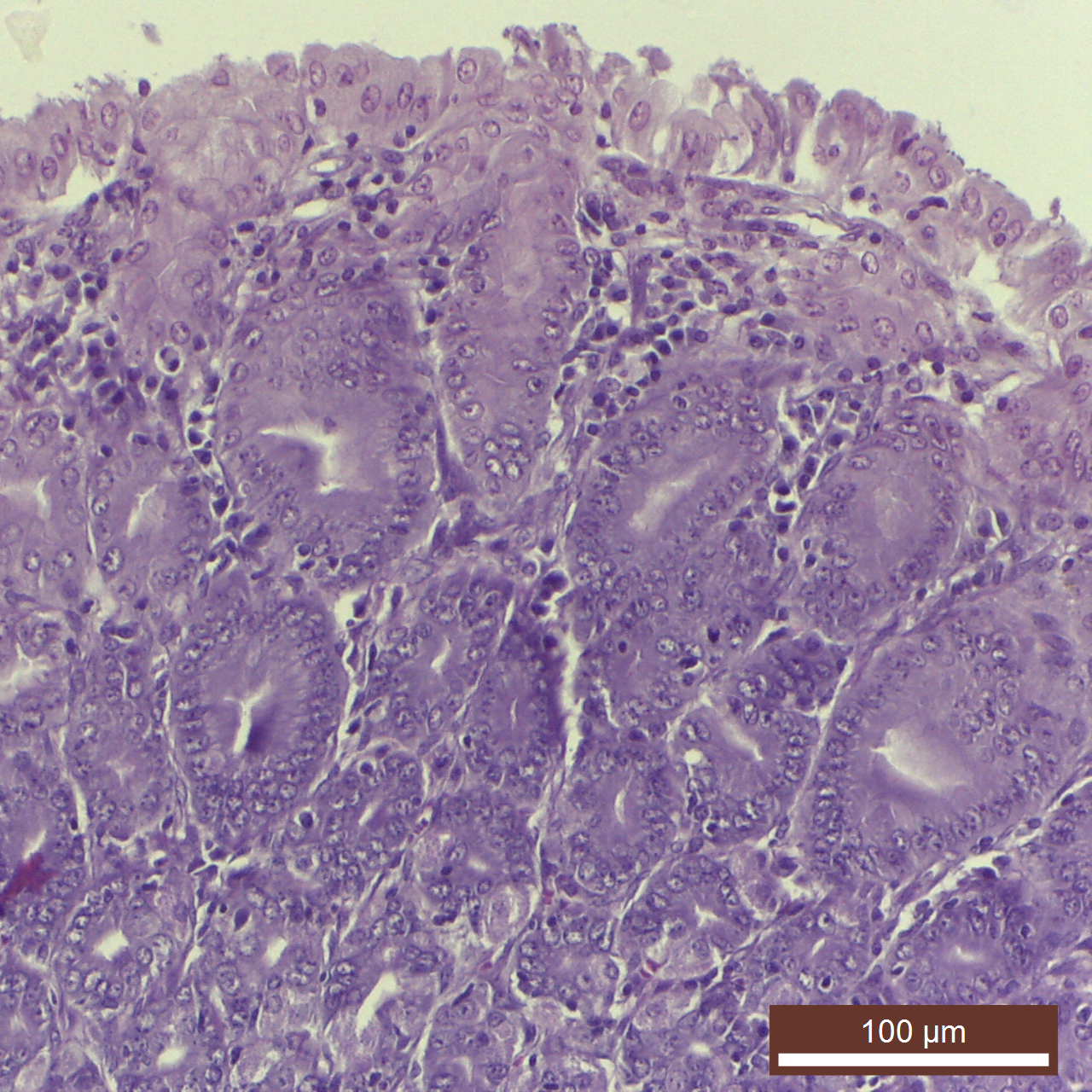

Supplement: Supplementary file 1 [file ijms-22-11534-s001.zip › Figure-S3-gastric-mucosa-secretory.tif]

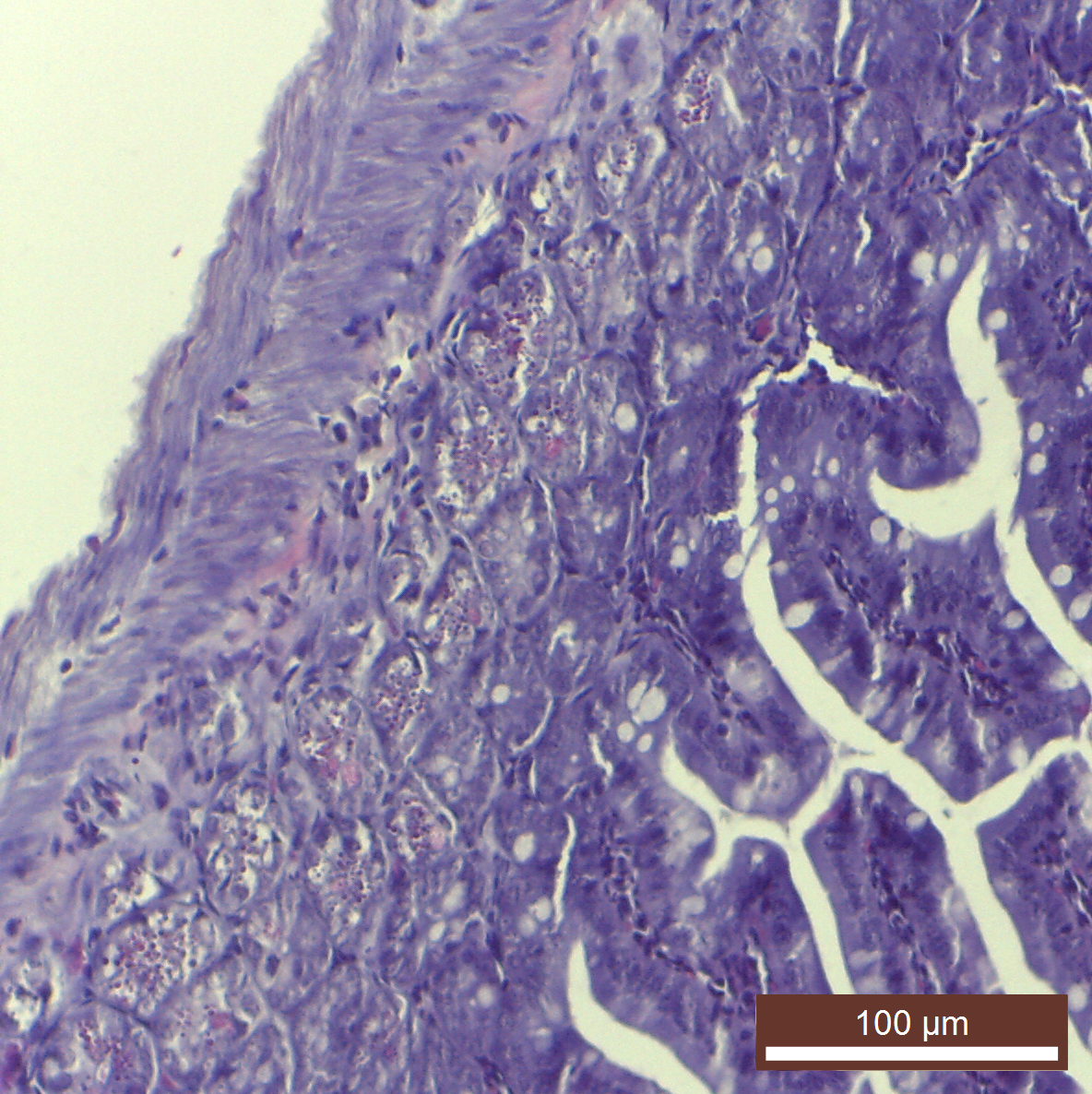

Supplement: Supplementary file 1 [file ijms-22-11534-s001.zip › Figure-S4-small-intestine-duodenum.tif]

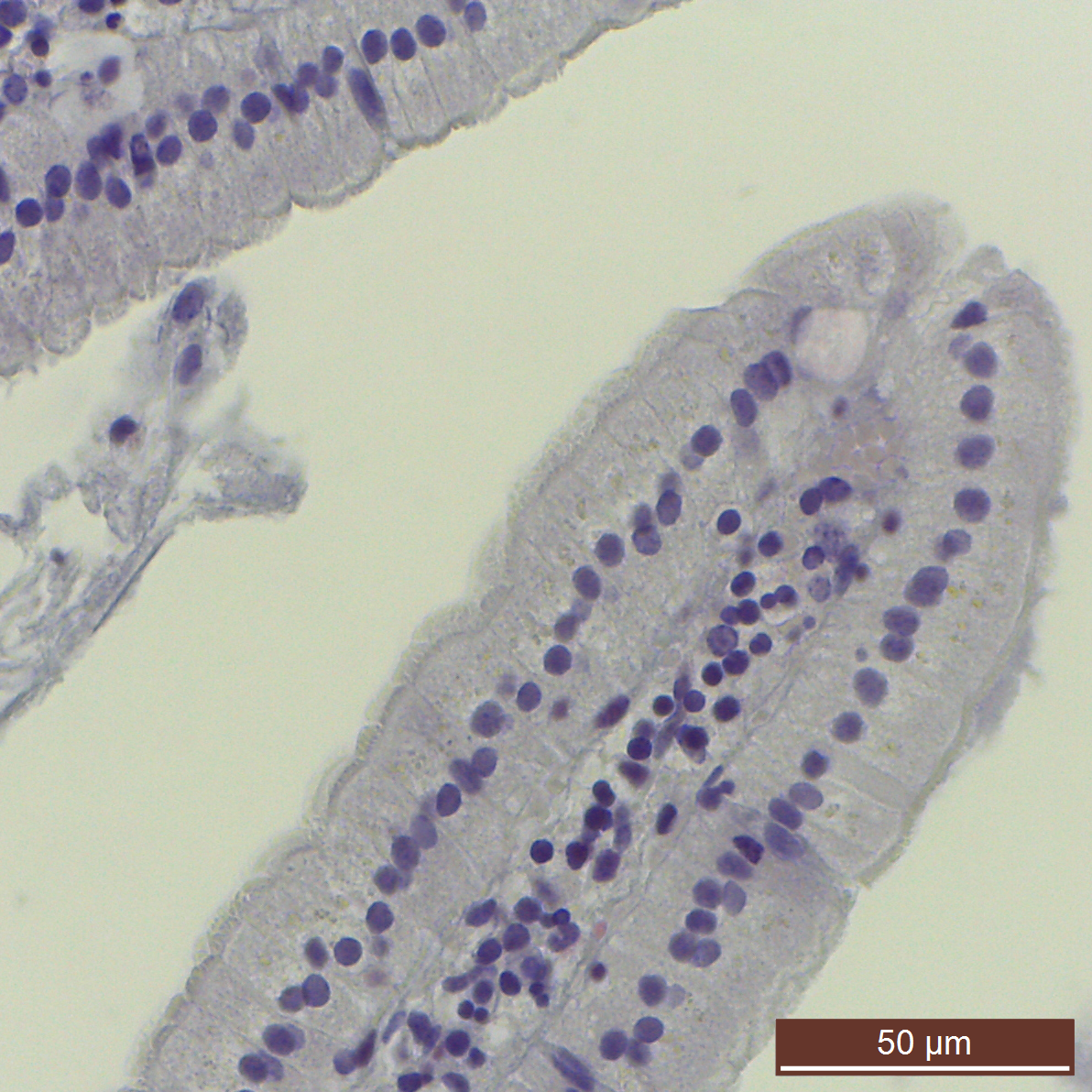

Supplement: Supplementary file 1 [file ijms-22-11534-s001.zip › Figure-S5-small-intestine-jejunum.tif]

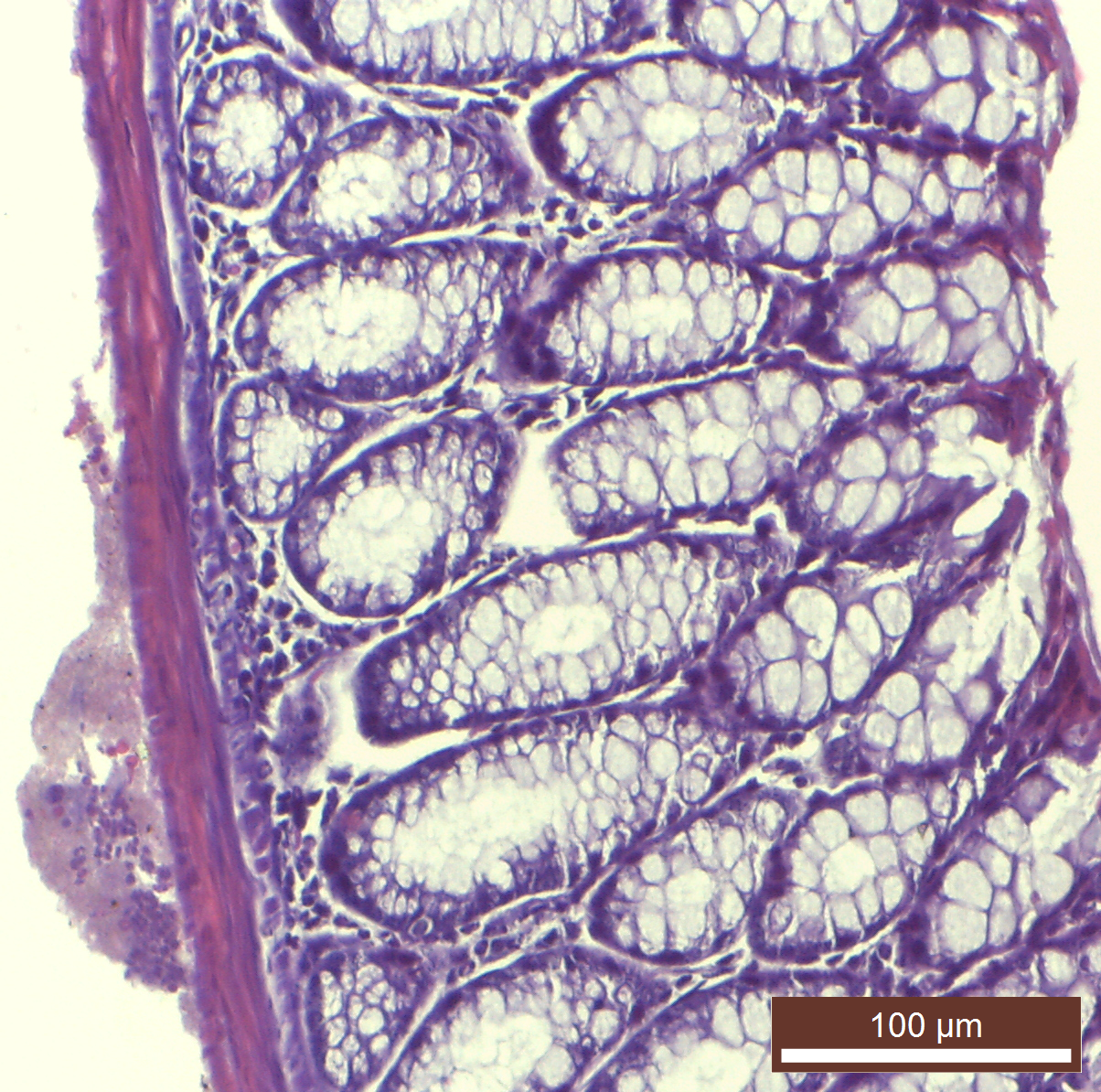

Supplement: Supplementary file 1 [file ijms-22-11534-s001.zip › Figure-S6-large-intestine-colon.tif]

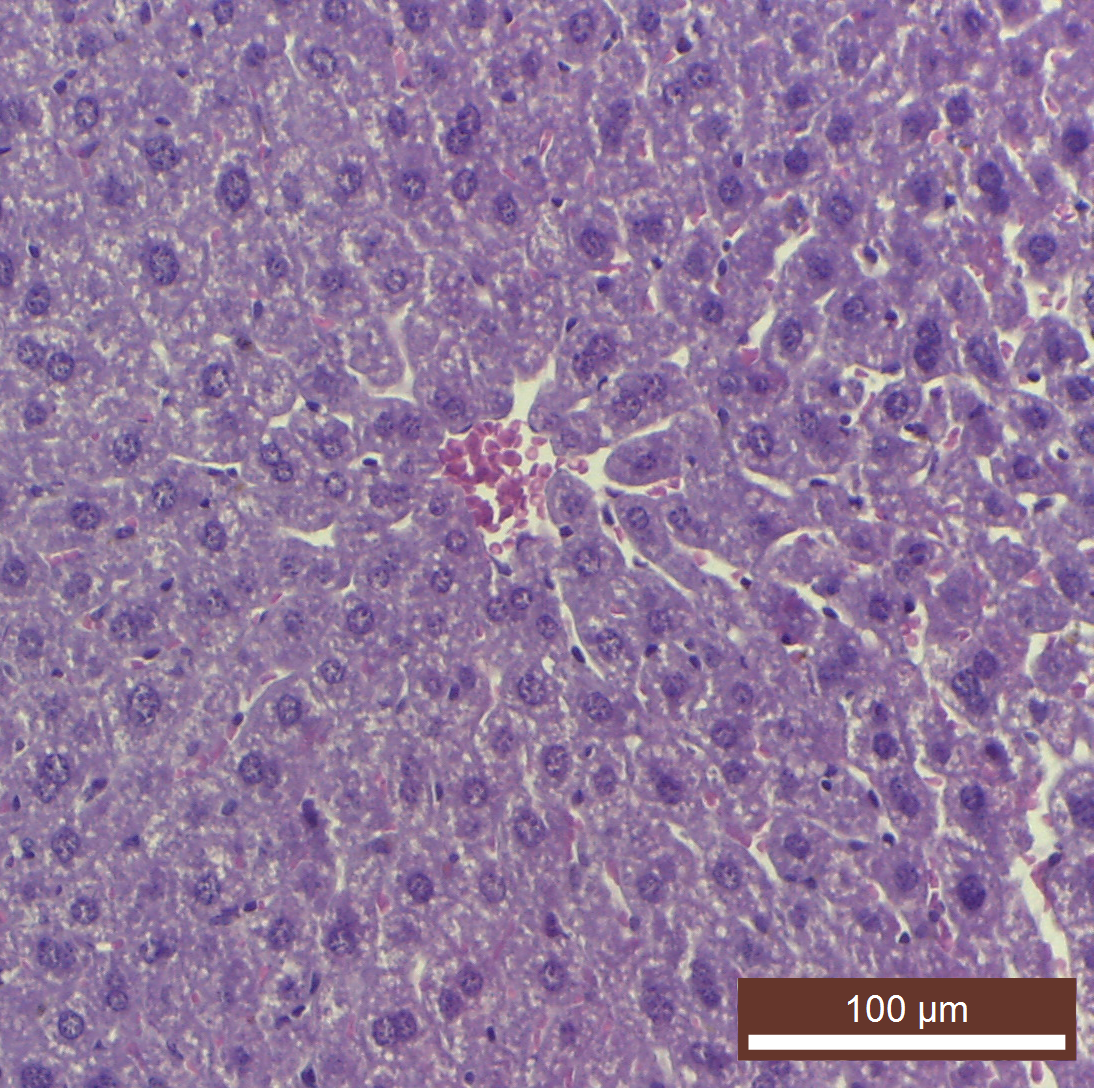

Supplement: Supplementary file 1 [file ijms-22-11534-s001.zip › Figure-S7-liver.tif]

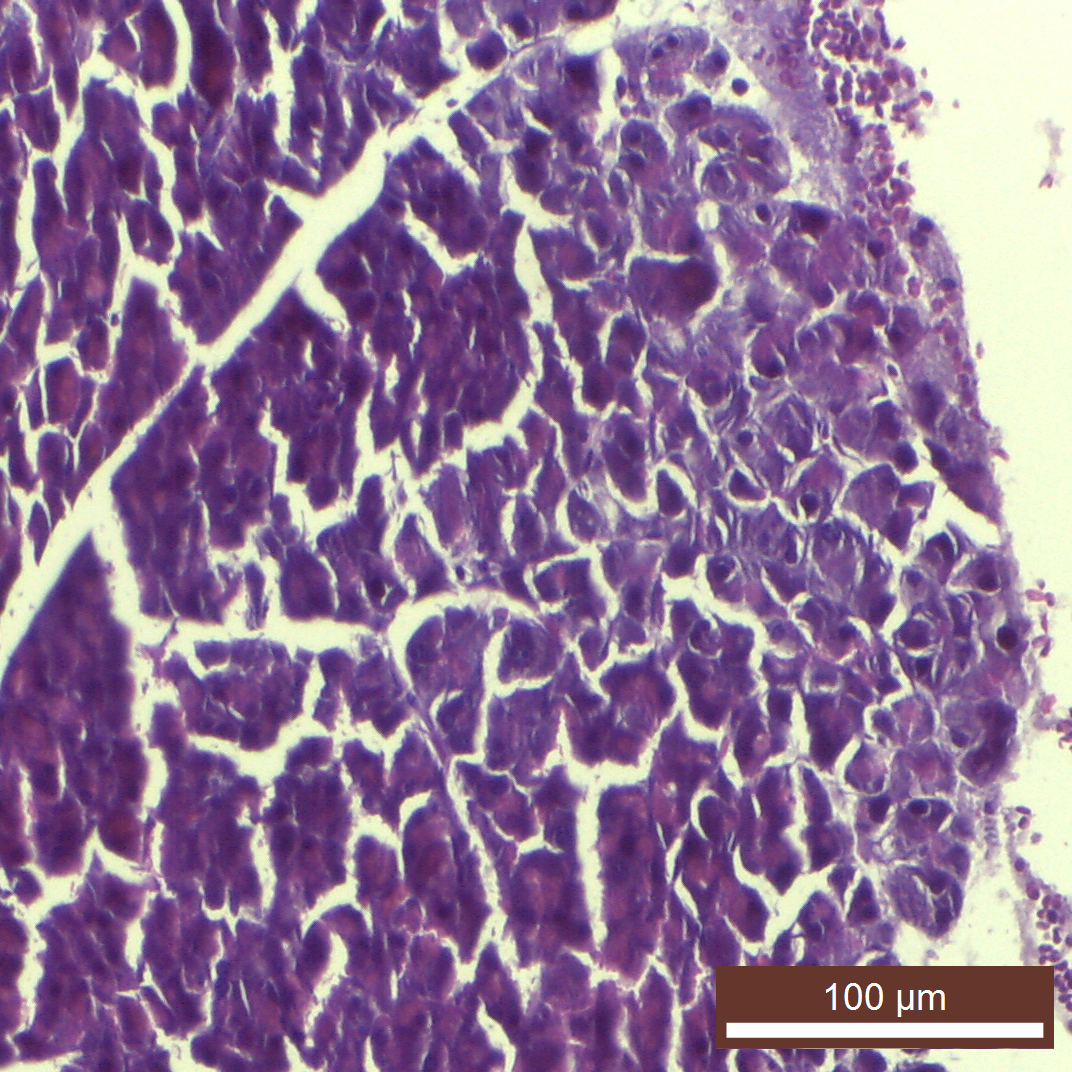

Supplement: Supplementary file 1 [file ijms-22-11534-s001.zip › Figure-S8-pancreas.tif]

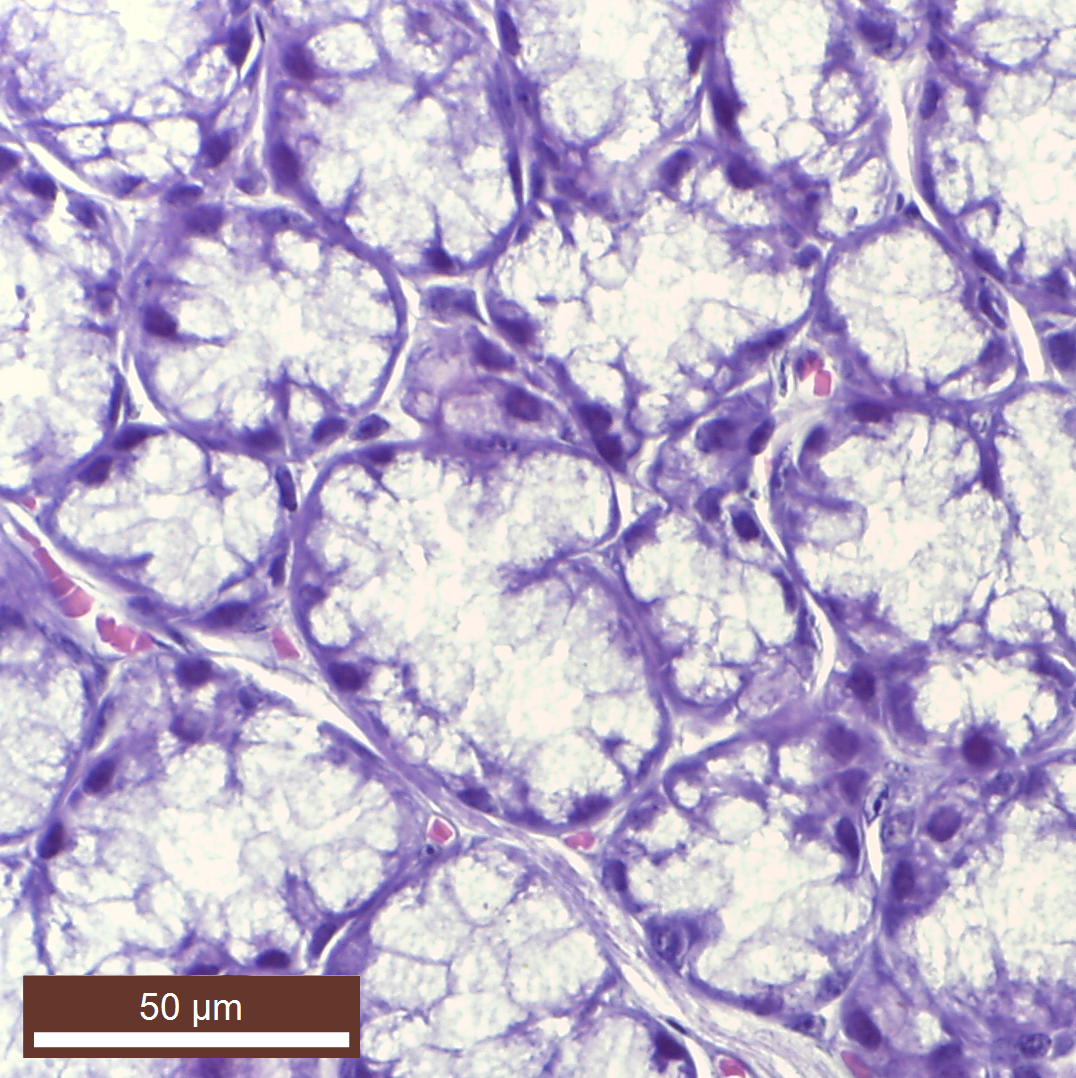

Supplement: Supplementary file 1 [file ijms-22-11534-s001.zip › Figure-S9-sublingual-glands.tif]
